# Molecular characterization of *TCF3*::*PBX1* chromosomal breakpoints in acute lymphoblastic leukemia and their use for measurable residual disease assessment

**DOI:** 10.1038/s41598-023-42294-9

**Published:** 2023-09-13

**Authors:** Thomas Burmeister, Daniela Gröger, Nicola Gökbuget, Bernd Spriewald, Michael Starck, Ahmet Elmaagacli, Dieter Hoelzer, Ulrich Keller, Stefan Schwartz

**Affiliations:** 1 Department of Hematology, Oncology and Tumor Immunology, CVK, Charité – Universitätsmedizin, corporate member of Freie Universität Berlin and Humboldt-Universität zu Berlin, Berlin, Germany; 2 Department of Hematology, Oncology and Tumor Immunology, CBF, Charité – Universitätsmedizin, corporate member of Freie Universität Berlin and Humboldt-Universität zu Berlin, Berlin, Germany; 3 Medical Department 2, Goethe-Universität, Frankfurt, Germany; 4 Department of Internal Medicine 5, Hematology and Oncology, University Hospital Erlangen, Erlangen, Germany; 5 I. Medical Department, München Klinik Schwabing, Munich, Germany; 6 Department of Hematology, Oncology, Asklepios Klinik St. Georg, Hamburg, Germany

**Keywords:** Cancer genetics, Molecular biology, Translational research, Acute lymphocytic leukaemia

## Abstract

The translocation t(1;19)(q23;p13) with the resulting chimeric *TCF3*::*PBX1* gene is the third most prevalent recurrent chromosomal translocation in acute lymphoblastic leukemia and accounts for 3–5% of cases. The molecular background of this translocation has been incompletely studied, especially in adult cases. We characterized the chromosomal breakpoints of 49 patients with *TCF3*::*PBX1* and the corresponding reciprocal *PBX1*::*TCF3* breakpoints in 15 cases at the molecular level, thus providing an extensive molecular overview of this translocation in a well-defined study patient population. Breakpoints were found to be remarkably clustered not only in *TCF3* but also in *PBX1*. No association with DNA repeats or putative cryptic recombination signal sequence sites was observed. A simplified detection method for breakpoint identification was developed and the feasibility of patient-specific chromosomal break sites as molecular markers for detecting measurable residual disease (MRD) was explored. A highly sensitive generic real-time PCR for MRD assessment using these breakpoint sequences was established that could serve as a useful alternative to the classical method utilizing rearranged immune gene loci. This study provides the first extensive molecular data set on the chromosomal breakpoints of the t(1;19)/*TCF3*::*PBX1* aberration in adult ALL. Based on the obtained data a generic MRD method was developed that has several theoretical advantages, including an on average higher sensitivity and a greater stability of the molecular marker in the course of disease.

## Introduction

The chromosomal translocation t(1;19)(q23;p13) with the formation of a chimeric *TCF3*::*PBX1* gene (*E2A-PBX1* in older nomenclature) is detected in approximately 3–5% of pediatric and adult acute lymphoblastic leukemia (ALL) cases. Despite its relative rarity, the translocation is still the third most frequent recurrent chromosomal translocation in ALL (after t(9;22)/*BCR*::*ABL1* and t(4;11)/*KMT2A*::*AFF1* in adult ALL and after t(12;21)/*ETV6*::*RUNX1* and t(4;11)/*KMT2A*::*AFF1* in pediatric ALL)^1^. Affected patients exhibit a characteristic B-cell immunophenotype (CD19+/CD10+/CD33−/CD34−/sIg−, and mostly cyIg+), and gene expression analyses have indicated that *TCF3*::*PBX1*-positive patients constitute a separate entity among ALL patients^2–4^. The current WHO classification includes *TCF3*::*PBX1*-positive ALL as a distinct subgroup of B-lymphoblastic leukemia^5^. Historically, *TCF3*::*PBX1*-positive leukemia has been associated with a poor prognosis, but this has been overcome by modern therapy regimens and *TCF3*::*PBX1* currently defines a group of ALL patients with a good clinical outcome in childhood ALL^16,6–9^, although these patients appear to have an increased risk for CNS involvement at diagnosis^10^. The prognostic impact of *TCF3*::*PBX1* in ALL in older patients (age > 15 years) is less well defined, and relatively few molecular-based and controversial data have been published in this area^11–15^. *TCF3*::*PBX1*-positive ALL has been found to express the receptor tyrosine kinase ROR1, which may serve as a therapeutic target in the future^16,17^. Some promising therapeutic in vitro effects have been observed with the SRC inhibitor dasatinib^18^ and the phosphatidylinositide 3-kinase delta (p110δ) inhibitor idelalisib^19^. However, no established targeted therapy currently exists for *TCF3*::*PBX1*-positive patients, and the assessment of measurable residual disease (MRD) remains the most important tool in therapy stratification and prognostication.

Data on the molecular details of the t(1;19)(q23;p13) translocation in adult ALL are scant. The following work analyzed 49 *TCF3*::*PBX1*-positive, clinically well-defined adult cases, identified the chromosomal break sites, and characterized the molecular background of this translocation, thus providing a detailed and extensive molecular overview of this translocation. A method for the easy identification of the breakpoint sites is presented, and the potential utilization of these chromosomal breakpoints for detecting measurable residual disease is demonstrated.

## Results

### Rationale for use and development of a long range-inverse PCR method

**Figure 1:**
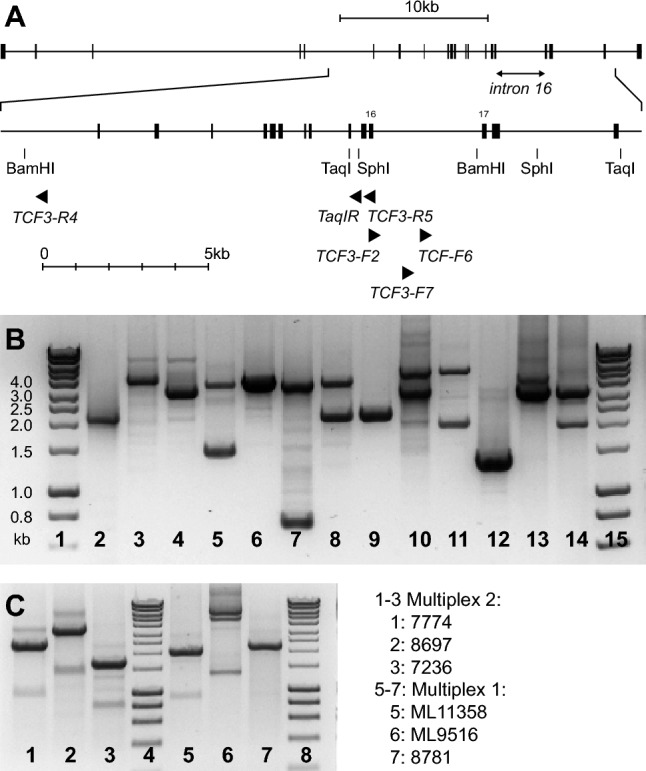
(**A**) Schematic depiction of the breakpoint region in *TCF3* with restriction sites, (**B**) Examples of long-range inverse PCR products (lanes 2–4: *Taq*I, lanes 5–12 *Sph*I, lanes 13–14 *Bam*HI, lanes 1 and 15: 1 kb Hyperladder. (**C**) Examples of long-range multiplex PCR results (with sample numbers).

One type of chimeric RNA transcript was predominantly found in *TCF3*::*PBX1*-positive patients, showing a fusion of *TCF3* exon 16 (reference sequence NG_029953.2) and *PBX1* exon 3 (reference sequence NG_028246.2)^26^. Other transcripts have been described, but they seem to be very rare^27^. Chromosomal breaks can thus be assumed to occur in the intron 3′ of *TCF3* exon 16 (“intron 16”) and in the intron 5′ of *PBX1* exon 3 (“intron 2”). The *TCF3* reference sequence includes a 3289 bp intron 16 (ncl 1615822–1619110, NC_000019.10, GRCh38.p13 primary assembly). This intron is present in all 41 *TCF3* variants listed in the NCBI gene database (updated on 1-Aug-2020). The location of the breakpoint site on chromosome 1 is less clear (8 *PBX1* transcript variants with either a 229,182 bp intron 2 (ncl 164563312–164792493) or a 166,397 bp intron 2 (ncl 164626097–164792493). Since the breakpoint region on chromosome 19 appeared to be relatively localized, a long range-inverse PCR (LRI PCR) approach was chosen for the analysis. Commercially available restriction enzymes were screened for those with restriction sites flanking the putative breakpoint region on chromosome 19. Three enzymes were suitable because they had palindromic cutting sites without degenerate nucleotides, produced sticky ends and were frequent cutters: *Sph*I, *Bam*HI and *Taq*I (Fig. 1A). *Bam*HI had one cutting site 148 bp 5′ of the *TCF3* intron end; thus, breakpoints near the intron end could not be detected using this enzyme. The three enzymes provided dense coverage of *PBX1* intron 2 with restriction sites (Table S1 and Figure S1).

Various PCR primers and PCR conditions were tested for the development of the inverse PCR method. The efficacy of inverse PCR could be optimized because the three enzymes produced a detectable “control” PCR product when testing normal DNA. The final PCR primer locations are depicted in Fig. 1A.

### Analysis of patient samples and chromosomal breakage data

**Figure 2:**
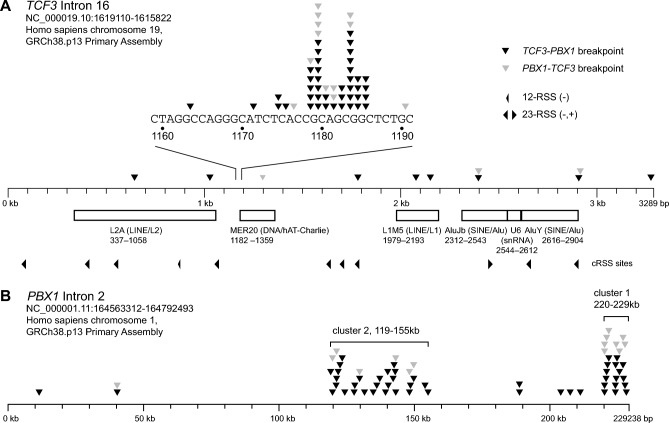
(**A**) Breakpoint distribution and location of DNA repeats/cRSS sites in *TCF3* intron 16, (**B**) breakpoint distribution in *PBX1* intron 2.

Patient samples were first analyzed with *Sph*I, followed by *Bam*HI and *Taq*I. Twenty-six *TCF3*::*PBX1* breakpoints were identified with *Sph*I, 12 with *Bam*HI and 11 with *Taq*I. PCR examples are shown in Fig. 1B. Fifty four *TCF3*::*PBX1*-RT-PCR-positive samples were analyzed and the genomic *TCF3*::*PBX1* fusion site was identified in 49 cases. The fact that five samples remained uncharacterized on the genomic level may reflect a limitation of the method. Insufficient DNA quality may also have played a role since some samples were more than 15 years old. Of these 49 *TCF3*::*PBX1* sites, the reciprocal *PBX1*::*TCF3* breakpoint was identified in 15 cases. One sample (5741) harbored an inversion with two breaks in *PBX1*. The break sites displayed a remarkable nonrandom pattern (Fig. 2, Table 1). Fifty-three (83%; 41 *TCF3*::*PBX1*, 12 *PBX1*::*TCF3*) were located in a narrow 40 bp region in *TCF3* intron 16, while 11 (17%; 8 *TCF3*::*PBX1*, 3 *PBX1*::*TCF3*) occurred outside this region. In *PBX1* intron 2 the clustering was less dense but still apparent. Two major break clusters could be delineated in *PBX1*: one from ~ 220 to 229 kb (near the intron end), and a second from ~ 120 to ~ 155 kb. These two clusters comprised 34 kb (14.8% of the intron) and 88% of all break events (Fig. 2, Table 1). Twenty-four breaks (37%; 16 *TCF3*::*PBX1*, 8 *PBX1*::*TCF3*) were located in the first cluster, 33 (51%; 27 *TCF3*::*PBX1*, 6 *PBX1*::*TCF3*) in the second cluster, and 8 (12%; 7 *TCF3*::*PBX1*, 1 *PBX1*::*TCF3*) outside both clusters.

**Table 1:** Basic characteristics of patient samples and breakpoint locations.

ID	*TCF3*::*PBX1*		*PBX1*::*TCF3*		Sample	Sex	Age	Phenotype	GenBank/ENA/DDBJ accession number(s)
	*TCF3*	*PBX1*	*TCF3*	*PBX1*					
2895	1163	40,275	1297	40,249	pb	f	30	pre B	OK334233, OK334275
3069	1180	140,360	–	–	bm	m	19	common	ON809522
3120	1179	127,732	–	–	bm	m	19	pre B	OK334234
3596	1182	121,859	–	–	bm	m	50	pre B	OK334235
3766	1178	228,226	–	–	pb	m	17	pre B	OK334236
3878	1782	221,288	–	–	bm	f	27	pre B	OK334237
3951	2151	11,457	–	–	bm	f	24	pre B	OK334238
3999	1180	188,792	–	–	bm	f	19	pre B	OK334239
4167	1183	142,143	–	–	bm	f	57	pre B	OK334240
4297	1179	220,178	1178	220,176	pb	f	24	pre B	OK334241, OK334276
4574	1174	227,739	1181	227,723	bm	m	28	pre B	ON383218, ON383219
4641	1185	188,751	–	–	pb	m	68	pre B	ON383220
4946	1179	155,038	–	–	pb	m	21	pre B	OK334242
5077	1178	224,786	–	–	bm	m	28	pre B	ON383221
5341	2908	220,185	2914	220,183	bm	m	31	pre B	OK334244, OK334277
5489	1179	224,501	1190	224,493	pb	f	39	pre B	OK334245, OK334278
5741^†^	1179	122,528/ 123,344	–	–	bm	f	49	pre B	OK334246
5752	1181	204,015	–	–	bm	f	64	pre B	OK334247
5850	1183	136,101	–	–	n.a	m	26	pre B	OK334248
5974	1174	131,565	–	–	pb	m	54	common	OK334249
6017	1183	153,909	–	–	pb	f	33	pre B	ON383222
6255	1183	225,679	1179	225,678	bm	m	43	pre B	OK334250, OK334279
6610	1184	224,282	–	–	pb	f	38	pre B	ON383223
6776	1183	139,327	–	–	pb	f	42	pre B	OK334251
6840	1028	227,189	–	–	pb	f	34	common	OK334252
7236	1175	124,284	–	–	bm	m	43	pre B	OK334253
7281	644	119,075	–	–	bm	m	36	pre B	ON383224
7431	1184	207,507	–	–	bm	f	47	pre B	OK334254
7533	1183	143,100	1179	143,096	bm	f	49	common	OK334255, OK334280
7601	1182	220,952	1183	220,955	bm	f	69	pre B	OK334256, OK334281
7613	1178	227,012	1181	227,012	bm	m	58	pre B	OK334257, OK334282
7911	1185	142,906	–	–	bm	m	36	pre B	OK334258
7979	1179	221,756	1181	221,756	bm	f	62	pre B	OK334259
8697	1171	137,203	–	–	bm	f	45	common	OK334260, OK334283
8781	2078	224,147	–	–	bm	m	58	pre B	OK334261
ML2764	1183	121,081	–	–	pb	f	64	pre B	OK334262
ML4316	1179	148,164	1176	148,145	pb	f	61	pre B	OK334263, OK334284
ML4863	1178	142,494	–	–	pb	f	40	pre B	OK334264
ML5319	1185	134,787	–	–	bm	m	77	pre B	OK334243
ML5947	1184	121,262	1179	121,248	pb	f	52	pre B	OK334265, OK334285
ML7774	2396	149,737	2397	149,723	pb	f	41	pre B	OK334266, OK334286
ML9516	1182	224,181	–	–	bm	m	67	pre B	OK334267
ML9735	3275	148,531	–	–	bm	f	22	pre B	OK334268
ML10287	1185	119,745	1183	119,741	bm	m	24	pre B	OK334269, OK334287
ML11220	1178	127,984	–	–	bm	m	67	pre B	OK334270
ML11358	1183	211,236	–	–	pb	f	19	pre B	OK334271
ML11543	1179	129,745	1179	129,741	bm	m	46	pre B	OK334272, OK334288
ML13676	1183	220,775	–	–	bm	f	31	common	OK334273
ML13772	1184	140,140	–	–	bm	f	32	pre B	OK334274

†Sample 5741 contained a 788 bp inversion in *PBX1* and thus two breaks in *PBX1*.

The break sites were analyzed bioinformatically for patterns that could explain the observed distribution. There were no apparent DNA microhomologies at the break sites in *PBX1* (Table S2). *TCF3* intron 16 included 6 DNA repeats comprising 1699 bp (51.6% of the intron), and *PBX1* intron 2 included 342 DNA repeats with 51,699 bp (22.6%, Tables S4, S5). The break cluster in *TCF3* intron 16 was located at the 5′ end of a repetitive DNA element (MER20). Eight of the 10 break sites in *TCF3* outside the cluster were located in or immediately 3′ of other DNA repetitive elements (L2A, MER20, L1MS, Alujb, AluY; Fig. 2A, Tables 1, S6). For the breaks in *PBX1*, there was no apparent association with repetitive DNA elements. Eight of the 55 breaks in *PBX1* (14.5%) occurred inside repetitive elements (# 3120, 3951, 5741, 5974, 6840, 7236, ML4316, ML11220; Tables 1, S4). Bioinformatical analysis revealed 611 potential cryptic recombination signal sequences (cRSSs) in *PBX1* intron 2, covering 20.1 kb (8.8%) of the intron. Five of the 65 breaks in *PBX1* (7.7%) occurred in or in the vicinity (± 30 bp) of cRSSs (# 3120, 4641, 7281, ML11220, ML11543; Tables 1, S7).

To complement this analysis, all samples were also investigated for intragenic *IKZF1* deletions by PCR. These deletions are found in approximately 20% of B-cell precursor ALL cases and are known to be caused by aberrant VDJ recombinase activity. None of the 49 samples showed an intragenic *IKZF1* deletion. This does not exclude a possible role of RAG-mediated secondary aberrations in *TCF3*::*PBX1*-rearranged ALL as illustrated by the example of *ETV6*::*RUNX1*-positive pediatric ALL^286,29^.

Chromosomal translocations are occasionally associated with DNA secondary structures, such as inverted repeats with hairpin loops^30^, and thus, the hotspot region of *TCF3* was analyzed with *RNAfold*. The main break site was located in an open loop that was flanked by regions with relatively strong base pair binding (Fig. S8). The analysis of the 15 cases in which reciprocal *PBX1*::*TCF3* were characterized showed mostly no microhomologies at the break sites, with frequent insertion of nontemplate nucleotides, suggesting a nonhomologous end-joining repair (NHEJ) mechanism^31^. One sample (4297) showed an insertion from the *FGF6* gene on chromosome 12, a gene not previously implicated in the pathogenesis of ALL (Fig. S3).

### Development and optimization of a real-time qPCR method

**Figure 3:**
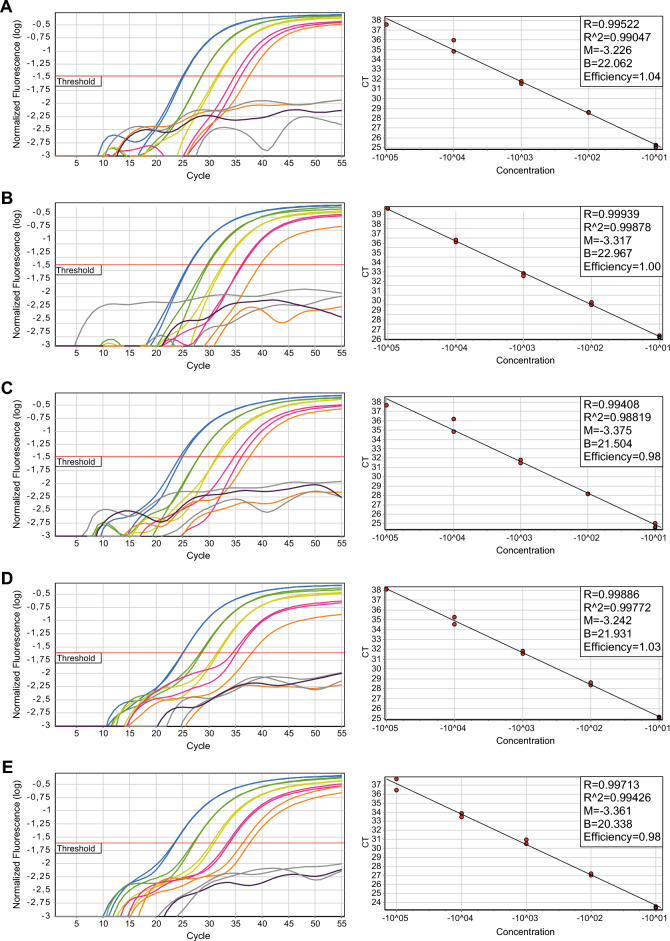
Examples of real time quantitative PCRs: (**A**) sample 4316, (**B**) sample 6255, (**C**) sample 7601, (**D**) sample 7979, (**E**) sample ML11543.

The clustering of chromosomal breaks in a narrow region in *TCF3* intron 16 suggested a quantitative PCR method with a common forward primer, a common dual-labeled hybridization probe 5′ of the breakpoint cluster region and a patient-specific reverse primer 3′ of the breakpoint. Several forward primers and dual-labeled probes were first tested on patient samples and control DNA to exclude spurious amplifications. Finally, one combination of a common forward primer and a common dual-labelled probe was selected that was tested on 15 randomly chosen patient samples (Table 2, Fig. 3). In all 15 cases, it was possible to design a reverse PCR primer that yielded data with good sensitivity and specificity. The testing of further samples was not possible because of shortage of sample material. This generic real-time qPCR was designed to quantify breakpoints in the *TCF3* hotspot region (~ 80%). Breakpoints outside this region (and likewise the *PBX1*::*TCF3* breakpoints) could theoretically also be used as MRD targets, but in these cases, no generic recipe can be given, and individual patient-specific qPCRs would have to be constructed.

**Table 2:** Real-time quantitative PCR parameters.

Patient	∆12	∆23	∆34	∆45	slope	R2	Efficiency
3766	3.52	3.28	3.60	3.19	− 3.418	0.999	0.961
4946	3.24	3.39	3.30	3.43	− 3.334	0.995	0.995
6255	3.44	3.01	3.53	3.37	− 3.317	0.999	1.002
7236	3.53	3.16	3.37	2.13	− 3.160	0.993	1.072
7533	3.26	3.37	3.10	3.41	− 3.257	0.979	1.028
7601	3.37	3.44	3.89	2.15	− 3.375	0.988	0.978
7979	3.40	3.18	3.20	3.22	− 3.242	0.998	1.035
ML2764	3.41	3.02	2.88	2.82	− 3.039	0.996	1.133
ML4316	3.45	3.05	3.76	2.16	− 3.226	0.990	1.042
ML4863	3.49	3.24	3.47	2.96	− 3.302	0.997	1.008
ML9516	3.54	3.41	2.55	2.73	− 3.042	0.994	1.132
ML11220	3.38	3.55	3.03	2.42	− 3.118	0.993	1.093
ML11358	3.33	3.31	3.01	2.91	− 3.144	0.989	1.080
ML11543	3.62	3.61	2.93	3.38	− 3.361	0.994	0.984
ML13772	3.59	3.57	3.17	–	− 3.456	0.994	0.947

The table shows the slopes of the standard curves, the correlation coefficients (R2) of the standard curves and the difference in Ct values between successive dilutions in the standard curve.

### Development and optimization of two multiplex long range PCRs

Since breakpoint identification by long range-inverse PCR is an elaborate procedure and since the breakpoints showed clustering in certain regions, efforts were made to simplify the detection procedure. Two multiplex long-range PCRs with a series of PCR oligonucleotides covering the entire breakpoint regions were developed and optimized that allowed the detection of breakpoints in the two breakpoint cluster regions of *PBX1*. Examples of these multiplex long-range PCRs are shown in Fig. 1C.

## Discussion

The translocation t(1;19)(q23;p13) has been described as mostly unbalanced^156,32–34^. This is in accordance with the observation made in this study that in only 14 cases (33%) a reciprocal break site could be characterized.

### Clustering of breakpoints

Since the first description of the translocation t(1;19) as a recurrent aberration in ALL in 1984, research has largely focused on cytogenetic aspects of this aberration, and few investigations have been carried out in adult ALL^276,346,35^. Wiemels et al*.*^36^ first systematically investigated the translocation at a molecular level and described 24 cases from various pediatric ALL studies. The median age of the patients was 6.8 years, with only one patient being an adult > 18 years of age. A similar clustering of breakpoints was observed, and the authors speculated that aberrant VDJ recombinase activity might be involved. They identified a reciprocal breakpoint in 5 (21%) cases^36^.

In this study, no association of t(1;19) chromosomal breaks with repetitive DNA elements was found. While the location of the break cluster in *TCF3* intron 16 close to a MER20 element could be coincidental, there was no similar association of the breaks mapping to *PBX1* intron 2. Similarly, no direct association with cryptic RSS was observed. None of the 49 patient samples showed an intragenic *IKZF1* deletion—an aberration caused by illegitimate VDJ recombination-mediated deletion, and present in approximately 20% of *BCR*::*ABL1*-negative B precursor ALLs. Recently, Liu et al*.* analyzed the *TCF3* “fragile zone” and suggested that the initial *TCF3* breakage may arise at a CpG site. They found a statistically significant proximity of the activation-induced cytidine deaminase (AID) hotspot motifs WRC and WGCW near the *TCF3* breakpoints W = A or T, R = A or G) suggesting AID involvement in the break process ^37^. This is consistent with the fact that *TCF3*::*PBX1* is predominantly detected in pre-B ALL, which is immunophenotypically the most “mature” entity in B precursor ALL, indicating a relatively late stage of B-cell development.

### Real-time qPCR for measurable residual disease detection

Measurable residual disease in ALL is usually assessed by the use of clonally rearranged immunoglobulin (IG) and/or T-cell receptor (TCR) loci for the construction of real-time quantitative PCRs (qPCRs)^38^. The main advantage of this approach is its universal applicability. Theoretically, it can be applied in any malignant disease of lymphatic origin. However, this method also has some disadvantages. In a significant minority of cases, it is not possible to identify clonal rearrangements, and with the introduction of next generation sequencing techniques it has become apparent that IG/TCR rearrangements are often in fact polyclonal at diagnosis^39^. IG/TCR-based MRD monitoring is thus often based on only one of several clones, and such an analysis may miss the decisive clone. IG/TCR-based qPCRs frequently show a suboptimal sensitivity (below 10^–4^), because of the difficulty of constructing a specific PCR against a highly homologous background. In addition, the IG/TCR rearrangements are potentially unstable, and further rearrangements can occur without loss of the malignant cell phenotype, leading to false negative results.

In those cases where chromosomal translocations lead to the expression of a chimeric mRNA transcript, MRD monitoring can also be performed by the relative quantification of this transcript^40^. However, this approach has been widely discarded in ALL (with the exception of *BCR*::*ABL1*), because it only allows a quantification relative to a “housekeeping gene”, assumed to be stably expressed. “Dormant” tumor stem cells with low expression of the oncogene may escape detection by RT-PCR. This is exemplarily illustrated by the observation that in *BCR*::*ABL1*-positive ALL, only a limited correlation between *BCR*::*ABL1*-mRNA-based and IG/TCR-based MRD levels is found^41^. Additionally, RNA is relatively unstable and significantly more difficult to handle than DNA.

An alternative approach is targeting the breakpoint sites of chromosomal translocations to detect and monitor MRD by constructing patient-specific qPCR assays. These are stable molecular markers that cannot be lost in the course of disease because they are linked to molecular drivers of the disease. This approach has been exploited in various entities, such as ALL with t(12;21)/*ETV6*::*RUNX1*^426,43^, ALL with 11q23/*KMT2A* aberrations^446,45^, ALL or CML with t(9;22)/*BCR*::*ABL1*^416,46,47^ and other hematopoietic malignancies^48–50^. In most of these cases, it could be shown that the break site-specific PCRs were at least as reliable as the IG/TCR-based methods and yielded a superior sensitivity. The main disadvantages of this approach are the technical difficulties posed by the individual characterization of break sites which may in some cases be dispersed over hundreds of kilobases of genomic DNA, precluding this approach for routine clinical studies with the exception of *KMT2A*-rearranged ALL, where relatively standardized techniques for break site identification are in use^51^. With the increasing availability of next-generation sequencing techniques and their technical advances (e.g., nanopore sequencing or mate-pair sequencing) and better knowledge of the molecular background these difficulties are likely to be overcome in the future and MRD detection methods based on chromosomal breakpoints will become increasingly important^526,53^.

## Conclusions

The present work characterizes the t(1;19) chromosomal breakpoints of a large number of adult ALL patients from a well-defined study population and is the largest and the first major investigation on this topic in adult ALL. The results provide a representative and relatively unbiased overview of the molecular details of this aberration. Based on the experimental results, a simplified method for the rapid identification of chromosomal breakpoints is proposed and the usefulness of these chromosomal breakpoint data for measurable residual disease detection is demonstrated. While the theoretical advantages of such an MRD approach appear obvious, clinical studies are necessary to validate the *TCF3*::*PBX1* breakpoint fusion as MRD marker in a clinical context. Further testing and comparisons will have to be performed to fully establish *TCF3*::*PBX1* breakpoints as valuable MRD targets.

## Methods

### Patient samples and ethics statement

Patient samples were collected from residual diagnostic material obtained between 2001 and 2021 in the context of the German Multicenter ALL Therapy Studies (clinicaltrials.gov identifiers: 00199056 and 00198991). Patients gave written informed consent to scientific investigations on study inclusion and the studies were approved by local and central ethics committees, among them an ethics board of the Goethe University, Frankfurt/Main, Germany and the ethics board of the Charité Universitätsmedizin, Berlin, Germany. Our study complied with the principles set forth in the World Medical Association Declaration of Helsinki.

### Patient characteristics

Patient clinical details are summarized in Table 1. All patient samples included in this study (31 bone marrow, 17 peripheral blood, one unspecified) had been investigated by flow cytometry and RT-PCR at diagnosis. All patients exhibited a B precursor immunophenotype (CD19+/CD10+/CD33−/CD34−/sIg−). Forty-three (88%) showed a cyIg+ (pre B) and six a cyIg− (common) immunophenotype. All samples were tested negative for *BCR*::*ABL1* and positive for *TCF3*::*PBX1* by RT-PCR. Twenty-seven (55%) of the 49 patients were female and 22 male. The median age was 39.5 years (range 17–77 years).

### DNA isolation

DNA was isolated from archived or fresh samples using either the Gentra *PureGene* method (QIAGEN, Hilden, Germany), the *AllPrep DNA/RNA Kit* (QIAGEN) or in a few cases the DNA preparation from TRIzol (ThermoFisher Scientific, Darmstadt, Germany) with subsequent DNA purification.

### Long range-inverse PCR (LRI PCR)

The LRI PCR methods were developed and optimized for this study. The following restriction enzymes were used: *Sph*I (GCATG|C), *Bam*HI (G|GACC) and *Taq*I (T|CGA). *FastDigest* enzymes were used according to the manufacturer’s recommendations (ThermoFisher Scientific, Darmstadt, Germany). The conditions for the long range-inverse PCR were partially adopted from previous work^20^. Five hundred nanograms of genomic DNA was digested in a 50 µl volume, the reaction mix was inactivated, purified using the *MaXtract High Density kit* (QIAGEN, Hilden, Germany), ethanol-precipitated and dissolved in a final volume of 30 µl. The entire volume was used in the ligation procedure (50 µl final volume, 5 U T4 ligase, 16 °C overnight). After purification and ethanol precipitation as described above, the ligation mix was dissolved in 30 µl H_2_O. Five microliters was used in the long-range PCR with the *Expand Long Template PCR System kit* (Roche, Mannheim, Germany) with buffer 2 and the following cycler program: 95 °C 2 min, 15 cycles (94 °C 30 s, 65 °C 30 s, 68 °C 6 min), 20 cycles (94 °C 30 s, 65 °C 30 s, 68 °C 5 min with 20 s increment/cycle), and 68 °C 10 min, 4 °C. One enzyme-specific *reverse* (R) PCR primer was combined with a *forward* (F) primer. The following primer combinations were used: *Sph*I: *TCF3-F2*/*TCF3-R5*, *Bam*HI: *TCF3-F2*/*TCF3-R4*, *Taq*I: *TCF3-F2*/*TaqI-R*. If a PCR product was visible the PCR was repeated and primer *TCF3-F2* replaced by primers *TCF3-F7* or *TCF3-F6* to try to generate a smaller PCR product for easier sequencing. PCR products of interest were excised from the agarose gel, purified, and analyzed by Sanger sequencing.

### Oligonucleotide sequences for the long range-inverse PCR

All oligonucleotides were obtained from *tib molbiol* (Berlin, Germany). The LRI PCR primer sequences were (5′-3′): *TCF3-R4* GAAGGCCTGGGCTACGGAGGGGAACAGCT, *TCF3-F2* CTCCCTGACCTGTCTCGGCCTCCCGACT, *TCF3-F6* ACCTTGATTCTATCACTCCTAGGCCAGGGCA, *TCF3-R5* CACAGGCCTCCATTCATGTCCCTTCCGCA, *TaqI-R* AGGCCGTGGAGACCCCCGTCGTAGCT. Normal DNA (without t(1;19) translocation) generated “control bands” of the following sizes: *TCF3-F2*/*TCF3-R4* 3308 bp, *TCF3-F6*/*TCF3-R4* 2131 bp, *TCF3-F2*/*TCF3-R5* 5026 bp, *TCF3-F6*/*TCF3-R5* 3849 bp, *TCF3-F2*/*TaqI-R* 7567 bp, *TCF3-F6*/*TaqI-R* 6390 bp.

### Sanger sequencing

Apart from the oligonucleotides detailed above several ad hoc designed oligonucleotides were used for Sanger sequencing of individual samples. Technical Sanger sequencing of PCR products was performed by *Microsync SeqLab* (Göttingen, Germany). Analysis of chromatograms and sequence data assembly was performed at the Charité laboratory in Berlin.

### Sequence data

All nucleotide sequence data (91 328 bp) were submitted to the GenBank/ENA/DDBJ database and are available under the accession numbers OK334233-OK334288, ON383218-ON383224, ON809522.

### Real-time quantitative PCR

Real-time qPCR was performed on a RotorGene RG-3000 cycler (formerly Corbett Research, now subsidiary of QIAGEN, Hilden, Germany) using the *ABgene PCR QPCR Mix* (Thermo Fisher Scientific, Darmstadt, Germany). The forward primer *TCF3-qF* 5′-CAGGCAGACTTTCCAAGTACCTT-3′ was used with the dual-labelled probe *TCF3-FAM* 5′-6FAM-CTATCACTCCTAGGCCAGGGCATCT-BHQ1-3′ and a patient-specific reverse primer.

### Multiplex long range PCR

The two multiplex long range PCRs comprised the following oligonucleotides (100 nM of each oligonucleotide per reaction mix). For breakpoint cluster 1 (5′-3′): *TCF3-F7* AGGAGGGTTTCAGGCAGAGGGCGCA, *PBX-long1* CCCGGGGTTGTGCTTCCTCCACCCTT, *PBX-long2* TGCGCTCTCTCCCTCCCCCTCATCTCT, *PBX-long3* ACGTGGTCCTGCGAGGAGCTCTTAGA, *PBX-long4* TGCCCATGCAGCAGGTGACAAGGG, and for breakpoint cluster 2 (5′-3′): *TCF3-F7*, *PBX1-long5* ACGAATCAGGCAGCTGTACAGAAAGCA, *PBX1-long6* TCGGCCTCACCTAACTGACTTGCAGGT, *PBX1-long7* AGCACCATCCTGAAGTTGCTCGGCT, *PBX1-long8* TGCGGGAGGCTGGCAACATTGAGTC, *PBX1-long9* ACACAGGTGCTACCTCTGCTCTGCCA, *PBX1-long10* TCCAGCTACCTCATGGCTCGCTAGA. PCR conditions were the same as for the LRI PCR.

### PCR for *IKZF1* deletions

The main four intragenic *IKZF1* deletion variants Δ2–7, Δ2–8, Δ4–7, Δ4–8 were investigated by four different PCRs, and the intragenic *IKZF1* deletion variant Δ2–3 by one single RT-PCR as outlined previously^21^.

### Bioinformatics and software

Genomic repeats were analyzed with *RepeatMasker* version 4.0.9, *RSSSite* and the *Tandem repeats finder*^22–24^. DNA secondary structures were investigated using *RNAfold 2.4.18*^25^. Sanger sequence chromatograms were analyzed with *4Peaks* (Nucleobytes, Aalsmeer, The Netherlands) and Nucleotide BLAST (blastn) against the GRCh38.13 reference primary assembly human genome.

### Supplementary Information

Supplementary Information.

## Data Availability

All nucleotide sequences generated and analyzed during the current study are available in the GenBank/ENA/DDBJ database under the accession numbers OK334233-OK334288, ON383218-ON383224, ON809522.
