# Supplementary material for: Molecular characterization of TCF3::PBX1 chromosomal breakpoints in acute lymphoblastic leukemia and their use for measurable residual disease assessment
Source: Sci Rep. 2023 Sep 13;13:15167. doi: 10.1038/s41598-023-42294-9 (PMC10499895; doi:10.1038/s41598-023-42294-9)

Supplement

Table of contents

Figure S1 and Table S1: Restriction map of *PBX1* intron 2 and table of restriction sites	1
Table S2: Alignment of the *TCF3* hotspot region	2
Figure S3: Chromosomal breakpoint and repair patterns	3
Table S4: Repeats in *PBX1* intron 2	4
Table S5: Repeats in *TCF3* intron 16	9
Table S6: Potential cryptic recombination signal sequences in *PBX1* intron 2	10
Table S7: Potential cryptic recombination signal sequences in *TCF3* intron 16	20
Figure S8: Two-dimensional DNAfold model of the *TCF3* hotspot region	21
Figure S9: Original gel images from Figure 1	22

Figure S1 and Table S1: Restriction map of *PBX1* intron 2 and list of restriction sites

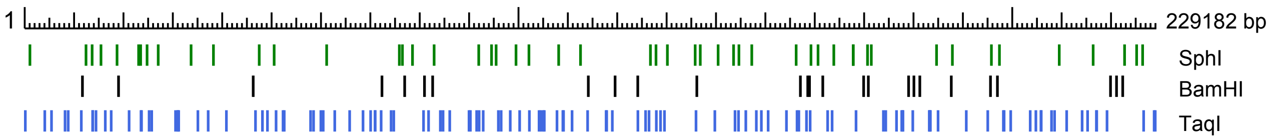

#	Site	Enzyme
1	128/130	TaqI
2	1058/1054	SphI
3	4066/4068	TaqI
4	5427/5429	TaqI
5	8174/8176	TaqI
6	8814/8816	TaqI
7	11467/11469	TaqI
8	11693/11697	BamHI
9	12415/12411	SphI
10	13655/13651	SphI
11	13772/13774	TaqI
12	13783/13785	TaqI
13	14440/14442	TaqI
14	15449/15445	SphI
15	16321/16323	TaqI
16	17518/17520	TaqI
17	18703/18699	SphI
18	19002/19006	BamHI
19	21143/21145	TaqI
20	23068/23064	SphI
21	23478/23474	SphI
22	23551/23553	TaqI
23	23659/23661	TaqI
24	24783/24779	SphI
25	25194/25196	TaqI
26	25724/25726	TaqI
27	27041/27037	SphI
28	30547/30549	TaqI
29	31021/31023	TaqI
30	31031/31033	TaqI
31	31190/31192	TaqI
32	33698/33694	SphI
33	35075/35077	TaqI
34	37156/37158	TaqI
35	38227/38223	SphI
36	40848/40850	TaqI
37	46277/46281	BamHI
38	46738/46740	TaqI
39	47495/47491	SphI
40	48147/48149	TaqI
41	49165/49167	TaqI
42	50518/50514	SphI
43	50909/50911	TaqI
44	52320/52322	TaqI
45	52561/52563	TaqI
46	57896/57898	TaqI
47	58503/58505	TaqI
48	59976/59978	TaqI
49	60458/60460	TaqI
50	61156/61152	SphI
51	62794/62796	TaqI
52	65808/65810	TaqI
53	68518/68520	TaqI
54	70057/70059	TaqI
55	70958/70960	TaqI
56	70965/70967	TaqI
57	72184/72186	TaqI
58	72355/72359	BamHI
59	74204/74206	TaqI
60	74745/74747	TaqI
61	75874/75870	SphI
62	76499/76495	SphI
63	76954/76958	BamHI
64	78535/78531	SphI
65	80769/80771	TaqI
66	80967/80971	BamHI
67	81792/81794	TaqI
68	82617/82621	BamHI
69	82914/82910	SphI
70	84173/84175	TaqI
71	84724/84726	TaqI
72	86080/86082	TaqI
73	89982/89984	TaqI
74	90258/90260	TaqI
75	91526/91528	TaqI
76	91950/91946	SphI
77	92028/92030	TaqI
78	92852/92854	TaqI
79	94546/94542	SphI
80	95471/95467	SphI
81	95928/95930	TaqI
82	96493/96495	TaqI
83	98334/98336	TaqI
84	99501/99497	SphI
85	100237/100239	TaqI
86	102121/102117	SphI
87	102208/102210	TaqI
88	104169/104171	TaqI
89	104535/104537	TaqI
90	105034/105036	TaqI
91	105231/105233	TaqI
92	107799/107801	TaqI
93	108139/108135	SphI
94	108987/108989	TaqI
95	109126/109128	TaqI
96	110850/110852	TaqI
97	112562/112558	SphI
98	114001/114003	TaqI
99	114144/114148	BamHI
100	117749/117751	TaqI
101	117865/117867	TaqI
102	119004/119006	TaqI
103	119564/119568	BamHI
104	124161/124165	BamHI
105	124184/124186	TaqI
106	125742/125744	TaqI
107	126441/126443	TaqI
108	126726/126722	SphI
109	127851/127847	SphI
110	127921/127923	TaqI
111	128767/128769	TaqI
112	129567/129569	TaqI
113	130158/130154	SphI
114	135784/135780	SphI
115	135955/135957	TaqI
116	136120/136124	BamHI
117	136819/136815	SphI
118	139769/139771	TaqI
119	140431/140427	SphI
120	143561/143557	SphI
121	143855/143857	TaqI
122	144562/144564	TaqI
123	144646/144642	SphI
124	146037/146039	TaqI
125	147248/147244	SphI
126	148172/148174	TaqI
127	149190/149192	TaqI
128	150566/150568	TaqI
129	154272/154274	TaqI
130	156237/156233	SphI
131	156416/156418	TaqI
132	156779/156781	TaqI
133	157093/157097	BamHI
134	158327/158329	TaqI
135	158586/158590	BamHI
136	158935/158939	BamHI
137	159068/159070	TaqI
138	159241/159237	SphI
139	160666/160662	SphI
140	161628/161632	BamHI
141	162615/162617	TaqI
142	163516/163518	TaqI
143	163859/163855	SphI
144	167786/167782	SphI
145	167790/167786	SphI
146	168362/168364	TaqI
147	169903/169907	BamHI
148	170689/170685	SphI
149	170853/170857	BamHI
150	171452/171448	SphI
151	173901/173903	TaqI
152	174386/174388	TaqI
153	176559/176561	TaqI
154	177615/177617	TaqI
155	177796/177798	TaqI
156	177864/177866	TaqI
157	179006/179010	BamHI
158	179855/179857	TaqI
159	180083/180087	BamHI
160	181280/181284	BamHI
161	183189/183191	TaqI
162	183284/183286	TaqI
163	183317/183319	TaqI
164	183418/183420	TaqI
165	184666/184662	SphI
166	184976/184978	TaqI
167	187877/187873	SphI
168	190690/190692	TaqI
169	195039/195041	TaqI
170	195776/195772	SphI
171	197404/197400	SphI
172	198201/198203	TaqI
173	198352/198354	TaqI
174	199688/199690	TaqI
175	203637/203639	TaqI
176	204782/204784	TaqI
177	205808/205810	TaqI
178	205868/205870	TaqI
179	207954/207956	TaqI
180	208571/208573	TaqI
181	209518/209514	SphI
182	211018/211020	TaqI
183	214148/214150	TaqI
184	215257/215259	TaqI
185	216385/216381	SphI
186	217037/217039	TaqI
187	217110/217112	TaqI
188	219194/219196	TaqI
189	222748/222744	SphI
190	225191/225187	SphI
191	226403/226399	SphI
192	226609/226611	TaqI
193	228732/228734	TaqI
194	228957/228959	TaqI

Table S2: Alignment of the *TCF3* hotspot region

ID	*TCF3*	filler sequence	*PBX1*
5974	TTCCAGGCAGACTTTCCAAGTACCTTGATTCTATCACTCCTAGGCCAGGGCATCT	GCGAAGGGGTCGGAGATGA	TTTCACCCATTCAGCAGTATTGACCTCTTGACTGGTGTCTCTGCCTGTAA
7236	TTCCAGGCAGACTTTCCAAGTACCTTGATTCTATCACTCCTAGGCCAGGGCATCTC	CC	GTGTCTAGCTTCTGGTCCACTTGTACTGGTGTTGACTATGTATCAGCCCA
ML11220	TTCCAGGCAGACTTTCCAAGTACCTTGATTCTATCACTCCTAGGCCAGGGCATCTCACC	CGACACGGAGGTGCTAA	GAGCCACTGTGCCCGGCGAGAGTTGTTTAATCTTGAAAAGTTGTCTAGCA
ML4863	TTCCAGGCAGACTTTCCAAGTACCTTGATTCTATCACTCCTAGGCCAGGGCATCTCACC	ATTACACTCTA	TTCAGAAGGTTTTCATGGTAGATACATTTAAAACATTCAAATAAGTTGTT
7613	TTCCAGGCAGACTTTCCAAGTACCTTGATTCTATCACTCCTAGGCCAGGGCATCTCACC	CCTTCCCCGGTCCCGAGTTACGCAA	GAAGGATTTTATTGGAAAAAAAAGTCCACAAAATAAACCTCAATGACCTC
3766	TTCCAGGCAGACTTTCCAAGTACCTTGATTCTATCACTCCTAGGCCAGGGCATCTCACC		ACAGGAAAGGTCTGAAGATTTCGTTCCACATGTACTGGATTTGTGTACTT
3120	TTCCAGGCAGACTTTCCAAGTACCTTGATTCTATCACTCCTAGGCCAGGGCATCTCACC	CC	CTCCCTTTGTCACCCAGGCTGGAGTGCAGTGGTGCAATCTCGGCTCACTG
ML11543	TTCCAGGCAGACTTTCCAAGTACCTTGATTCTATCACTCCTAGGCCAGGGCATCTCACCG	AACTATTAAGGCGGTGGG	GCCAAGGTCACTGTGTGTGCCCCACAGGCCTCTGCAGGAAGAAAGAGTTG
ML4316	TTCCAGGCAGACTTTCCAAGTACCTTGATTCTATCACTCCTAGGCCAGGGCATCTCACCG	ATATC	GATGGTCTCGATCTCCTGACCTCGTGATCCACATGCCTTGGCCTCCCAAA
4946	TTCCAGGCAGACTTTCCAAGTACCTTGATTCTATCACTCCTAGGCCAGGGCATCTCACCG	TCTTTTTTA	CTGCCAATTAAAGCTTCCAGTTGTATCACATTTAGGAACTTTGAGGACAT
4297	TTCCAGGCAGACTTTCCAAGTACCTTGATTCTATCACTCCTAGGCCAGGGCATCTCACCG	GAAGCCAGAAAGTTACGGCACGAC	AACTCTTAACTCTAAATCTATCCTGCTTTTTTATTCTTCCCAAAGGGATC
7979	TTCCAGGCAGACTTTCCAAGTACCTTGATTCTATCACTCCTAGGCCAGGGCATCTCACCG	GATAGCCCTCTAACGACAGCGGCCAGCGGTGCGGTGC	ACTACAGCTTCTCATCTGAAGAGGCCGCTCCAGGCCTACTGCAGCATCCA
5489	TTCCAGGCAGACTTTCCAAGTACCTTGATTCTATCACTCCTAGGCCAGGGCATCTCACCG	GGGGG	ACCTGTCTCAACTGGTCTCTTCCATAATTGGGTGGATTTGTGGGAAGGAA
5741	TTCCAGGCAGACTTTCCAAGTACCTTGATTCTATCACTCCTAGGCCAGGGCATCTCACCG	ACCCCCCTAGGTCCTTGATCCATTTTGAGTTAAGTTTTGATTAT<br>GCTATGAGGTAAGGAAGCAAATTTATTCTTTTGCATATGGAAAT<br>GCAGTTGTACACCATGTTCTTCATTTCCCTTTGTGTCTCCCATT<br>GATTCACTTCTGTGAACACGAGGAGGAATCCCTTCCCCATTCTA<br>TCATCCAACCTCTCCTTTCTACCTTCTGATACCTTAAAGTGCCT<br>TTCCTCCTGACCATTCTGGGGCTGCTGCCAAGGTAAGTTCTTAT<br>GACCCATTTGTTCCTGATGCCACCTCTGCTGCATCTGTCCTTGT<br>TTTCACTCTGTTTGATTCCCATCAAATGGGCCATGGAAGTTTCC<br>CAAGAAGCCACACCAAATAAAAATGACAGGTTTGGCAACAACAG<br>AGAAACCTGGTAAAATGTTGATATTTGACAGAGCAGAGAAACTG<br>GGAAATGGGTGAACAGTTTAGTCACAGATCAAAAACCACATACT<br>CACAGGTTCCAGGTGACCGAACACTTTCATCTGAAAAGAGTACA<br>GTGGTAATCACCATAATAATAAGGAACATCATTTAGTGAGCACC<br>TACTGGGTGTTAGGCCTTTACACATATCCCCTCATCTAATCCTT<br>GTAAGTTCCCCGCCCTGTAGGTAGCTCTCCCCTTAATAGATGAA<br>TAAATGGAGGGCTGGAGAAATTCAGCAGCATTGATTGCCAATGT<br>CATCAATAGTAAATATCTGAAGGAGGATTCAAACTCAGGTCTAA<br>CTCCAAGGCCCAGACTCTTCCCCTGGGTGGTGCTGCACTTTGTA<br>ACACTCACCACGGGGGAACAGGCACCTAATGTTCTCCAGCAGAA<br>GCTGCAAGAAAACCATGGTCTGAGGTTTTCTCCAACAGATCCTG<br>AACTCTCACTGCCAGGAGGGCGGCCTACTAATGTAAGACCA	AGGCATGGGTCAGGTACATCTTCAGCCATGGGTAAGTTTGTTCAACAAAT
3999	TTCCAGGCAGACTTTCCAAGTACCTTGATTCTATCACTCCTAGGCCAGGGCATCTCACCGC	CCCCCA	ATTTCCTTTCAAATGCCAAGGGAGATTTTAACGTGGCTACCAAGAAACAA
5752	TTCCAGGCAGACTTTCCAAGTACCTTGATTCTATCACTCCTAGGCCAGGGCATCTCACCGCA	ATACGTTCAGCGGGACGCATGTGG	ATGAGGGGCCCTGGGGTTCCACACAGGTCTCATGCTCACAAAGCCAGTGC
3596	TTCCAGGCAGACTTTCCAAGTACCTTGATTCTATCACTCCTAGGCCAGGGCATCTCACCGCAG		AGTCATACAAAAGCATCACTGGCAGATTCCACCAGTGTTTTATTCACACC
7601	TTCCAGGCAGACTTTCCAAGTACCTTGATTCTATCACTCCTAGGCCAGGGCATCTCACCGCAG	TGACCC	GTTTCCCGAGCAGCAGTCCAGACTTGAGCTATTAAGGACCAAAGCCCCTC
ML9516	TTCCAGGCAGACTTTCCAAGTACCTTGATTCTATCACTCCTAGGCCAGGGCATCTCACCGCAG	ACAGGCTCTGTTTCAGTCCCCGGA	CCCTCTAGTGGGCATTGGCTGGAGCTGCTCCGGACCCACGTCACTCAGCC
ML2764	TTCCAGGCAGACTTTCCAAGTACCTTGATTCTATCACTCCTAGGCCAGGGCATCTCACCGCAGC	CG	ACTGAGGGAGATAAAAGGCAATCTATGCTTTACTGCTTTCCCTAAGTTTT
5850	TTCCAGGCAGACTTTCCAAGTACCTTGATTCTATCACTCCTAGGCCAGGGCATCTCACCGCAGC	AGTGGAGACAGGT	CCCGAGAACAAAGATTGTGGATCCCCAAAGTACCTCCACCTCCTCACTGG
6776	TTCCAGGCAGACTTTCCAAGTACCTTGATTCTATCACTCCTAGGCCAGGGCATCTCACCGCAGC	CCC	CAGTGCAGGAAATATCAGCCCTTTTCTAAAGCACTATGAAAATGAAAGGG
4167	TTCCAGGCAGACTTTCCAAGTACCTTGATTCTATCACTCCTAGGCCAGGGCATCTCACCGCAGC	TCTTTTTTTTTCGGAACACACCTTCGTCTCGTTACTTTATTGTC<br>TCCACTTTTCGCGTTTTTGCCGAATAAACGCGGTACCCCTACGA<br>TAAA	CAGCTCTTTTTTTTTCGGAACACACCTTCGTCTCGTTACTTTATTGTCTC
7533	TTCCAGGCAGACTTTCCAAGTACCTTGATTCTATCACTCCTAGGCCAGGGCATCTCACCGCAGC	ATTTACCCATC	TATCCATTGTAATCCTCCTAGTAAGTCATAAGATCTATTCACAATCACCC
ML11358	TTCCAGGCAGACTTTCCAAGTACCTTGATTCTATCACTCCTAGGCCAGGGCATCTCACCGCAGC	CCGGTGCAG	GTGTACTACAGACAATGCTGTAATTGCTTCTGTGACCAAGTGAGGGCGGA
6255	TTCCAGGCAGACTTTCCAAGTACCTTGATTCTATCACTCCTAGGCCAGGGCATCTCACCGCAGC	ACTCCTAGGCCCCCAAAAAAA	CTAAGCGAAATACTAACACTTTCTTGCCCCAGCCAACCCACCACCACTGC
ML5947	TTCCAGGCAGACTTTCCAAGTACCTTGATTCTATCACTCCTAGGCCAGGGCATCTCACCGCAGCG	AGGGAGCCCCC	ATATTTCTCTGAGTGTTCTAGATTCATAGACCCAAATGACCTCTCTGAGC
7431	TTCCAGGCAGACTTTCCAAGTACCTTGATTCTATCACTCCTAGGCCAGGGCATCTCACCGCAGCG	CCCCCCCAGACGGAGGACGC	GACAGCCGTGACCTCTGAAAGAGAGGCATAGGTCAGTAAAAGATGCATTC
ML13772	TTCCAGGCAGACTTTCCAAGTACCTTGATTCTATCACTCCTAGGCCAGGGCATCTCACCGCAGCG	ACGGTGAG	ATGGATTACTGGGTATAGGTCCATGTATTGTTGATCTGTGGCATGACAGG
ML5319	TTCCAGGCAGACTTTCCAAGTACCTTGATTCTATCACTCCTAGGCCAGGGCATCTCACCGCAGCGG	AGATAT	AGTGCCTGTATCTCCCAGGGCACCGATTGTTAAGAGGGTTGGGACCTCAG
7911	TTCCAGGCAGACTTTCCAAGTACCTTGATTCTATCACTCCTAGGCCAGGGCATCTCACCGCAGCGG	GTCGGTCCC	GTGGGTCACTGGGCTCCTCTGGAAACTTCTGTAGGCAGGGCAGAGGTTTC
ML10287	TTCCAGGCAGACTTTCCAAGTACCTTGATTCTATCACTCCTAGGCCAGGGCATCTCACCGCAGCGG	TAGGAAAGACGGGGAACC	GATTCTACAACCATCATCAAATTTTGGTTGAAAACTCAAAGCTGCTGATA

Figure S3: Chromosomal breakpoint and repair patterns

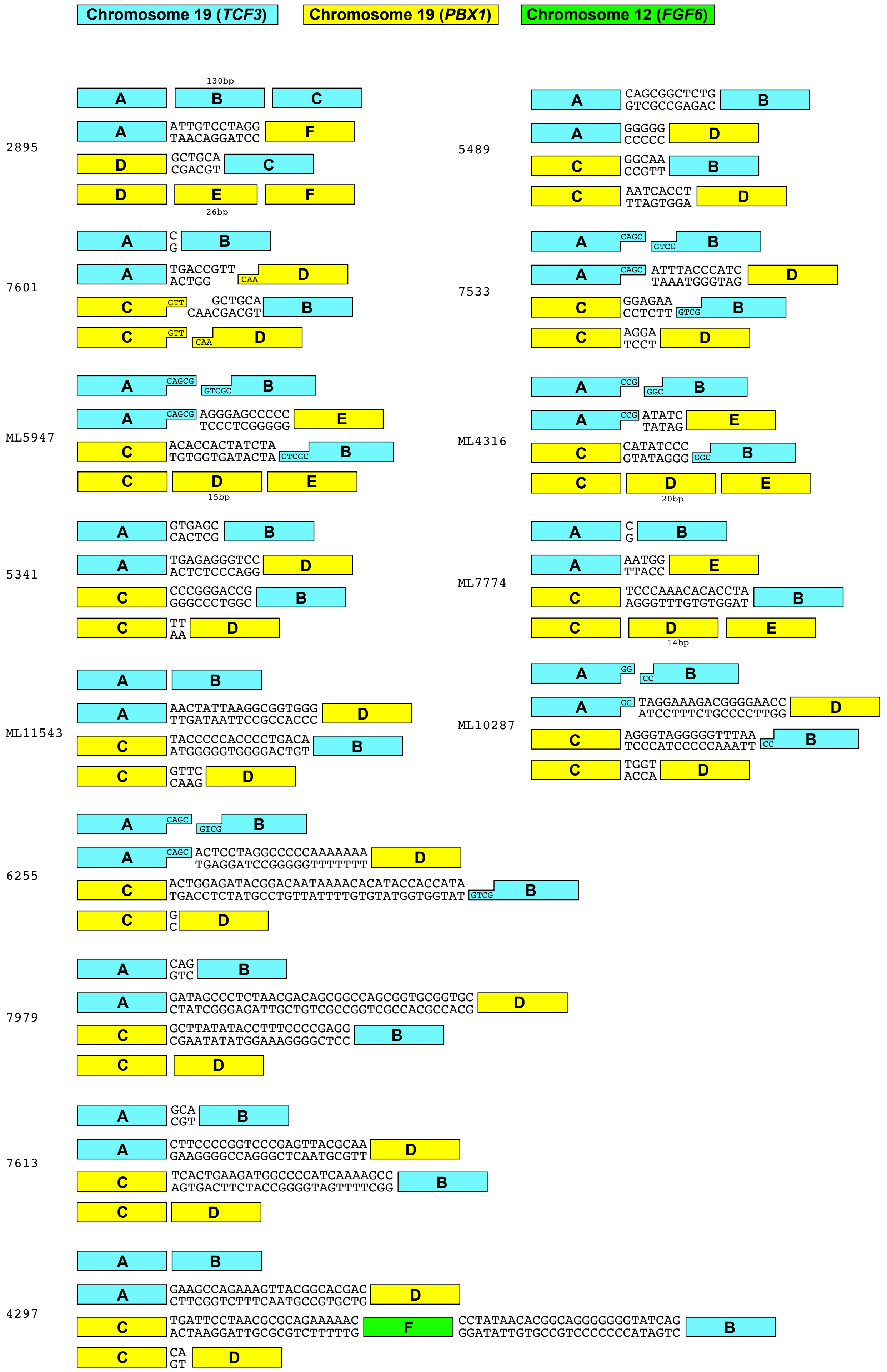

Table S4: Repeats in *PBX1* intron 2

#	query	%div.	%del.	%ins.	begin	end	orientation	repeat	class/family
1	PBX1 intron 2	0.0	4.8	0.0	947	967	+	(TATT)n	Simple repeat
2	PBX1 intron 2	15.5	0.0	0.0	1505	1533	+	(TA)n	Simple repeat
3	PBX1 intron 2	0.0	0.0	0.0	2112	2142	+	(AC)n	Simple repeat
4	PBX1 intron 2	20.5	0.0	0.0	3852	3893	+	(TG)n	Simple repeat
5	PBX1 intron 2	23.6	3.2	0.4	5858	6108	+	MIR	SINE/MIR
6	PBX1 intron 2	13.4	4.9	1.0	6236	6546	-	AluSx	SINE/Alu
7	PBX1 intron 2	6.4	0.3	0.3	6899	7763	-	L1PA7	LINE/L1
8	PBX1 intron 2	17.9	2.3	7.3	7853	8498	-	L1MB7	LINE/L1
9	PBX1 intron 2	9.8	0.0	3.9	8508	8560	-	L1MA10	LINE/L1
10	PBX1 intron 2	0.0	0.0	0.0	8563	8605	+	(TA)n	Simple repeat
11	PBX1 intron 2	12.5	3.5	0.0	8608	8895	-	AluSx	SINE/Alu
12	PBX1 intron 2	17.7	22.8	0.0	9132	9210	-	MamTip2	DNA/hAT-Tip100
13	PBX1 intron 2	34.5	4.4	0.7	9315	9451	+	MIRb	SINE/MIR
14	PBX1 intron 2	8.7	6.8	2.2	9771	9946	-	Charlie5	DNA/hAT-Charlie
15	PBX1 intron 2	14.8	3.0	9.0	9958	10173	-	MER33	DNA/hAT-Charlie
16	PBX1 intron 2	8.7	2.7	0.7	10174	10473	-	AluSx	SINE/Alu
17	PBX1 intron 2	15.7	2.6	7.9	10474	10574	-	MER33	DNA/hAT-Charlie
18	PBX1 intron 2	27.6	5.7	10.6	10946	11098	+	MIRc	SINE/MIR
19	PBX1 intron 2	9.4	2.4	0.0	11390	11677	+	AluSx	SINE/Alu
20	PBX1 intron 2	38.5	1.9	0.0	15183	15286	-	L2d2	LINE/L2
21	PBX1 intron 2	23.9	0.0	1.1	16556	16648	-	MIR	SINE/MIR
22	PBX1 intron 2	8.4	0.0	0.7	16972	17269	-	AluSx	SINE/Alu
23	PBX1 intron 2	11.3	0.0	1.0	17273	17566	-	AluSx	SINE/Alu
24	PBX1 intron 2	7.7	0.7	0.0	17918	18214	-	AluSx	SINE/Alu
25	PBX1 intron 2	12.5	2.6	5.3	18653	18691	+	(TTAT)n	Simple repeat
26	PBX1 intron 2	25.7	2.0	6.0	18924	19109	-	L4 A Mam	LINE/RTE-X
27	PBX1 intron 2	9.3	0.0	0.7	19110	19412	-	AluSx	SINE/Alu
28	PBX1 intron 2	25.7	2.0	6.0	19413	19469	-	L4 A Mam	LINE/RTE-X
29	PBX1 intron 2	35.0	10.3	1.6	19489	19662	+	L2c	LINE/L2
30	PBX1 intron 2	34.2	5.6	3.7	19708	19868	-	L2c	LINE/L2
31	PBX1 intron 2	22.9	2.2	19.8	20665	20800	+	L2c	LINE/L2
32	PBX1 intron 2	19.5	0.0	3.7	20907	20991	+	7SLRNA	srpRNA
33	PBX1 intron 2	26.1	0.0	0.0	20935	21026	-	SVA A	Retroposon/SVA
34	PBX1 intron 2	3.0	0.0	0.0	21040	21073	+	(GA)n	Simple repeat
35	PBX1 intron 2	7.7	3.3	3.3	21701	21730	+	(TAA)n	Simple repeat
36	PBX1 intron 2	0.0	0.0	0.0	22641	22666	+	(CATT)n	Simple repeat
37	PBX1 intron 2	24.2	5.7	10.1	23205	23328	+	MIRc	SINE/MIR
38	PBX1 intron 2	26.7	3.9	2.9	24017	24120	+	MIR3	SINE/MIR
39	PBX1 intron 2	0.0	0.0	0.0	25163	25189	+	(T)n	Simple repeat
40	PBX1 intron 2	17.4	2.9	2.9	25830	25864	+	GA-rich	Low complexity
41	PBX1 intron 2	24.0	2.5	4.5	26151	26307	+	MER135	DNA
42	PBX1 intron 2	32.8	0.0	0.0	26463	26523	-	MIR3	SINE/MIR
43	PBX1 intron 2	16.0	0.7	1.7	26616	26913	+	AluJb	SINE/Alu
44	PBX1 intron 2	15.7	0.0	0.0	27535	27700	-	AluSx	SINE/Alu
45	PBX1 intron 2	10.0	0.3	0.0	27701	28000	-	AluSx	SINE/Alu
46	PBX1 intron 2	27.0	8.3	3.0	28058	28405	+	MamRep434	DNA/TcMar-Tigger
47	PBX1 intron 2	13.0	0.0	0.7	29644	29946	-	AluSx	SINE/Alu
48	PBX1 intron 2	32.0	4.4	2.9	30173	30307	+	MIRb	SINE/MIR
49	PBX1 intron 2	19.3	19.1	0.9	30759	30847	-	MIRb	SINE/MIR
50	PBX1 intron 2	25.0	0.0	3.9	30913	30966	-	MIR3	SINE/MIR
51	PBX1 intron 2	8.9	5.0	0.3	30987	31269	-	AluSx	SINE/Alu
52	PBX1 intron 2	14.1	14.1	0.0	31300	31548	-	MER33	DNA/hAT-Charlie
53	PBX1 intron 2	18.7	0.0	5.1	31549	31611	-	MIR3	SINE/MIR
54	PBX1 intron 2	27.3	2.4	4.0	32803	32928	+	MamRep1151	LTR
55	PBX1 intron 2	12.0	4.0	1.3	33131	33206	+	MER20	DNA/hAT-Charlie
56	PBX1 intron 2	27.4	8.2	0.0	33372	33444	+	MIRc	SINE/MIR

57	PBX1 intron 2	24.7	1.1	1.1	33586	33679	-	MIR1 Amn	SINE/MIR
58	PBX1 intron 2	21.3	1.8	5.7	34494	34548	+	(AATT)n	Simple repeat
59	PBX1 intron 2	21.0	5.5	3.5	34602	34932	-	MSTD	LTR/ERVL-MaLR
60	PBX1 intron 2	20.7	2.1	2.1	34970	35017	+	(TTCTCTT)n	Simple repeat
61	PBX1 intron 2	23.4	5.4	3.5	35018	35025	+	(TTCC)n	Simple repeat
62	PBX1 intron 2	29.5	10.4	0.0	35507	35699	-	MIRc	SINE/MIR
63	PBX1 intron 2	29.6	0.0	0.0	35732	35777	+	(TT)n	Simple repeat
64	PBX1 intron 2	34.4	0.8	0.8	36753	36875	-	MER94B	DNA/hAT-Blackjack
65	PBX1 intron 2	8.9	1.0	0.0	36936	37237	-	AluSx	SINE/Alu
66	PBX1 intron 2	30.4	0.7	2.0	37352	37502	+	MamSINE1	SINE/tRNA-RTE
67	PBX1 intron 2	8.2	1.0	0.7	37644	37951	+	AluSx	SINE/Alu
68	PBX1 intron 2	15.8	4.5	2.2	40469	40512	+	(TTTTAAG)n	Simple repeat
69	PBX1 intron 2	34.1	5.8	2.2	40542	40618	-	MIR	SINE/MIR
70	PBX1 intron 2	0.0	0.0	0.0	40619	40651	+	(T)n	Simple repeat
71	PBX1 intron 2	13.5	0.4	0.4	40662	40929	-	AluSx	SINE/Alu
72	PBX1 intron 2	34.1	5.8	2.2	40930	41014	-	MIR	SINE/MIR
73	PBX1 intron 2	10.8	0.0	12.8	41121	41173	-	L2c	LINE/L2
74	PBX1 intron 2	35.6	6.2	4.1	41707	41946	-	L2c	LINE/L2
75	PBX1 intron 2	27.1	12.4	4.6	42411	42693	+	MamRep434	DNA/TcMar-Tigger
76	PBX1 intron 2	13.9	0.0	0.0	42897	43126	+	AluSx	SINE/Alu
77	PBX1 intron 2	15.3	0.0	9.2	43143	43272	+	AluSx	SINE/Alu
78	PBX1 intron 2	22.1	7.1	0.0	43314	43453	+	L2a	LINE/L2
79	PBX1 intron 2	33.6	7.8	1.6	44050	44524	-	L2c	LINE/L2
80	PBX1 intron 2	19.5	0.0	0.0	44698	44738	+	MER117	DNA/hAT-Charlie
81	PBX1 intron 2	29.6	3.4	3.4	44738	44856	+	MER117	DNA/hAT-Charlie
82	PBX1 intron 2	17.7	1.6	0.0	44873	44934	-	L2b	LINE/L2
83	PBX1 intron 2	25.4	1.7	1.7	44899	44958	+	L2a	LINE/L2
84	PBX1 intron 2	31.7	0.0	0.0	45210	45291	-	MIRc	SINE/MIR
85	PBX1 intron 2	27.5	0.0	2.0	46434	46537	+	L2b	LINE/L2
86	PBX1 intron 2	3.0	0.0	0.3	47927	48226	-	AluY	SINE/Alu
87	PBX1 intron 2	28.6	7.1	0.0	49498	49707	+	MIR	SINE/MIR
88	PBX1 intron 2	18.4	16.7	4.1	49754	49861	-	L1ME4b	LINE/L1
89	PBX1 intron 2	35.8	7.3	1.3	50008	50225	+	L2b	LINE/L2
90	PBX1 intron 2	32.0	2.6	2.6	50880	51033	-	MIR3	SINE/MIR
91	PBX1 intron 2	13.7	0.0	0.7	51462	51763	-	AluSx	SINE/Alu
92	PBX1 intron 2	28.2	1.2	1.7	53456	53697	-	MIR	SINE/MIR
93	PBX1 intron 2	27.2	11.4	1.1	53854	54098	+	MamSINE1	SINE/tRNA-RTE
94	PBX1 intron 2	27.1	9.4	4.5	54231	54400	+	MER103C	DNA/hAT-Charlie
95	PBX1 intron 2	31.5	2.1	5.7	56294	56438	-	Charlie4z	DNA/hAT-Charlie
96	PBX1 intron 2	13.7	5.8	6.9	56581	56871	+	AluJb	SINE/Alu
97	PBX1 intron 2	11.2	0.0	0.0	57366	57670	-	AluSx	SINE/Alu
98	PBX1 intron 2	9.6	1.1	0.0	57691	57971	-	AluSx	SINE/Alu
99	PBX1 intron 2	25.4	0.0	6.1	57995	58046	+	(TCTT)n	Simple repeat
100	PBX1 intron 2	25.7	0.0	2.1	59454	59502	+	(CA)n	Simple repeat
101	PBX1 intron 2	9.3	4.1	0.0	59512	59801	-	AluSx	SINE/Alu
102	PBX1 intron 2	27.7	2.3	6.8	61193	61544	+	Charlie23a	DNA/hAT-Charlie
103	PBX1 intron 2	9.3	0.0	0.0	62390	62412	+	(TG)n	Simple repeat
104	PBX1 intron 2	4.3	0.0	0.0	62499	62522	+	(A)n	Simple repeat
105	PBX1 intron 2	20.8	2.1	0.0	63256	63303	-	L3b	LINE/CR1
106	PBX1 intron 2	15.9	0.0	0.0	65400	65427	+	(TC)n	Simple repeat
107	PBX1 intron 2	20.9	9.0	1.4	65428	65694	-	UCON64	Unknown
108	PBX1 intron 2	34.3	3.9	1.1	66148	66327	-	MIR3	SINE/MIR
109	PBX1 intron 2	28.1	15.0	0.0	67301	67453	-	MIRb	SINE/MIR
110	PBX1 intron 2	4.5	0.0	0.0	67965	67987	+	(T)n	Simple repeat
111	PBX1 intron 2	29.9	12.4	0.0	69611	69747	-	MIR3	SINE/MIR
112	PBX1 intron 2	11.2	0.0	0.0	69835	70138	-	AluSx	SINE/Alu
113	PBX1 intron 2	24.4	5.5	2.7	70336	70588	+	MIR	SINE/MIR
114	PBX1 intron 2	12.0	3.7	0.0	73547	73573	+	(TG)n	Simple repeat
115	PBX1 intron 2	29.4	0.0	1.5	74124	74192	+	MIR3	SINE/MIR
116	PBX1 intron 2	10.7	0.0	0.0	75259	75288	+	(AC)n	Simple repeat

117	PBX1 intron 2	31.7	5.9	1.1	75685	75853	+	MIRb	SINE/MIR
118	PBX1 intron 2	17.3	0.0	0.0	76254	76355	+	U6	snRNA
119	PBX1 intron 2	18.7	0.3	0.3	76356	76650	-	AluJb	SINE/Alu
120	PBX1 intron 2	12.2	0.0	0.3	77235	77538	-	AluSx	SINE/Alu
121	PBX1 intron 2	20.5	5.0	0.0	78701	78740	+	(TTTTG)n	Simple repeat
122	PBX1 intron 2	27.4	0.0	0.0	79401	79473	-	L2d2	LINE/L2
123	PBX1 intron 2	33.5	5.4	2.2	80329	80551	-	MIRb	SINE/MIR
124	PBX1 intron 2	26.6	5.8	1.2	82565	82720	+	MIR1 Amn	SINE/MIR
125	PBX1 intron 2	27.2	12.3	0.7	84104	84225	+	MIR3	SINE/MIR
126	PBX1 intron 2	22.0	0.0	3.5	84358	84503	+	MER5B	DNA/hAT-Charlie
127	PBX1 intron 2	6.0	0.0	0.3	84505	84803	-	AluY	SINE/Alu
128	PBX1 intron 2	29.6	2.4	1.2	85199	85362	-	MIRb	SINE/MIR
129	PBX1 intron 2	14.5	1.0	0.0	86906	87209	-	AluSx	SINE/Alu
130	PBX1 intron 2	20.9	4.5	0.0	88246	88289	+	GA-rich	Low complexity
131	PBX1 intron 2	0.0	0.0	0.0	88620	88659	+	(GT)n	Simple repeat
132	PBX1 intron 2	11.6	2.2	0.0	88757	88980	-	AluSx	SINE/Alu
133	PBX1 intron 2	34.3	3.8	2.1	89077	89311	-	MIRc	SINE/MIR
134	PBX1 intron 2	23.7	12.7	3.0	89387	89562	-	L1ME2	LINE/L1
135	PBX1 intron 2	13.7	1.0	0.0	89563	89855	-	AluSx	SINE/Alu
136	PBX1 intron 2	23.7	12.7	3.0	89856	90039	-	L1ME2	LINE/L1
137	PBX1 intron 2	13.4	1.1	0.0	90180	90448	+	AluSx	SINE/Alu
138	PBX1 intron 2	11.6	0.3	0.3	91790	92101	-	AluSx	SINE/Alu
139	PBX1 intron 2	14.0	5.9	1.9	92679	92729	+	Mam R4	LINE/Dong-R4
140	PBX1 intron 2	13.1	8.0	0.0	93494	93553	+	(TTATA)n	Simple repeat
141	PBX1 intron 2	30.4	3.2	1.6	93744	94120	+	Mam R4	LINE/Dong-R4
142	PBX1 intron 2	5.6	0.0	0.0	94856	94874	+	(A)n	Simple repeat
143	PBX1 intron 2	28.2	0.0	0.0	95352	95436	+	MIRb	SINE/MIR
144	PBX1 intron 2	23.5	3.3	1.7	95436	95690	-	MIR	SINE/MIR
145	PBX1 intron 2	29.5	0.0	0.0	96612	96740	+	MLT1N2	LTR/ERVL-MaLR
146	PBX1 intron 2	9.3	0.0	0.0	98114	98415	-	AluSx	SINE/Alu
147	PBX1 intron 2	13.8	0.0	0.0	98736	99031	+	AluSx	SINE/Alu
148	PBX1 intron 2	18.6	0.0	1.8	99822	99998	+	(CCTT)n	Simple repeat
149	PBX1 intron 2	6.7	9.4	0.0	99999	100030	+	(CT)n	Simple repeat
150	PBX1 intron 2	18.6	0.0	1.8	100031	100053	+	(CCTT)n	Simple repeat
151	PBX1 intron 2	10.7	5.4	1.9	101303	101654	-	THE1B	LTR/ERVL-MaLR
152	PBX1 intron 2	15.4	0.0	0.6	101963	102275	-	AluSx	SINE/Alu
153	PBX1 intron 2	29.2	3.5	3.5	102534	102675	-	MLT1N2	LTR/ERVL-MaLR
154	PBX1 intron 2	4.9	0.0	0.0	103036	103056	+	(ATT)n	Simple repeat
155	PBX1 intron 2	24.2	0.0	14.5	103101	103187	+	Mam R4	LINE/Dong-R4
156	PBX1 intron 2	28.9	1.6	2.7	103210	103394	+	Mam R4	LINE/Dong-R4
157	PBX1 intron 2	33.8	4.3	0.5	103485	103695	-	MIRb	SINE/MIR
158	PBX1 intron 2	25.0	6.1	0.0	104021	104151	+	(AT)n	Simple repeat
159	PBX1 intron 2	26.4	10.6	0.5	104301	104479	+	Tigger12	DNA/TcMar-Tigger
160	PBX1 intron 2	20.1	2.9	0.0	106496	106650	-	L1M3	LINE/L1
161	PBX1 intron 2	32.6	0.0	0.0	108668	108739	+	(TTTT)n	Simple repeat
162	PBX1 intron 2	20.2	1.7	1.7	109597	109605	+	(TCTTTTT)n	Simple repeat
163	PBX1 intron 2	17.7	0.0	0.0	109609	109640	+	(T)n	Simple repeat
164	PBX1 intron 2	20.2	1.7	1.7	109643	109655	+	(TCTTTTT)n	Simple repeat
165	PBX1 intron 2	8.3	0.0	0.0	110155	110323	-	AluSx	SINE/Alu
166	PBX1 intron 2	15.4	5.1	0.0	110660	110698	-	MIR3	SINE/MIR
167	PBX1 intron 2	13.2	0.0	0.0	111537	111561	+	(C)n	Simple repeat
168	PBX1 intron 2	28.7	2.3	0.0	111616	111839	-	MIRc	SINE/MIR
169	PBX1 intron 2	6.4	1.3	0.0	112032	112109	+	MADE1	DNA/TcMar-Mariner
170	PBX1 intron 2	30.0	2.9	1.9	113308	113409	+	MIR	SINE/MIR
171	PBX1 intron 2	3.0	0.0	2.3	113773	114080	-	AluY	SINE/Alu
172	PBX1 intron 2	28.1	5.4	3.2	114134	114225	-	L2d2	LINE/L2
173	PBX1 intron 2	22.8	0.0	6.2	116476	116578	-	MIRc	SINE/MIR
174	PBX1 intron 2	15.6	0.0	1.0	117070	117166	-	L1PA16	LINE/L1
175	PBX1 intron 2	24.8	8.7	5.8	117379	117562	-	L3	LINE/CR1
176	PBX1 intron 2	16.4	9.6	0.4	117671	117878	+	AluJb	SINE/Alu

177	PBX1 intron 2	25.1	0.0	2.6	118020	118059	+	(TATACA)n	Simple repeat
178	PBX1 intron 2	13.8	0.0	0.0	118346	118533	+	L1PA15	LINE/L1
179	PBX1 intron 2	22.4	0.8	9.2	119088	119205	-	L2a	LINE/L2
180	PBX1 intron 2	34.1	9.4	0.0	119949	120086	-	MIRc	SINE/MIR
181	PBX1 intron 2	31.1	10.4	0.0	120087	120274	-	MIRc	SINE/MIR
182	PBX1 intron 2	18.1	4.6	4.6	120491	120806	-	AluJb	SINE/Alu
183	PBX1 intron 2	32.3	5.2	2.0	121654	121846	-	MIRc	SINE/MIR
184	PBX1 intron 2	29.5	9.2	5.4	122292	122542	-	MIRb	SINE/MIR
185	PBX1 intron 2	29.6	10.6	2.6	122647	122892	+	MIRb	SINE/MIR
186	PBX1 intron 2	7.0	0.4	0.7	123402	123676	+	AluY	SINE/Alu
187	PBX1 intron 2	33.8	4.6	8.1	123747	123969	+	MamRep38	DNA/hAT-Tip100
188	PBX1 intron 2	14.4	0.7	0.7	123970	124277	+	AluJb	SINE/Alu
189	PBX1 intron 2	33.8	4.6	8.1	124278	124333	+	MamRep38	DNA/hAT-Tip100
190	PBX1 intron 2	28.4	3.5	8.1	124920	125061	+	X6a DNA	DNA/TcMar-Tigger
191	PBX1 intron 2	18.4	0.0	0.0	125983	126033	+	(T)n	Simple repeat
192	PBX1 intron 2	20.7	0.0	2.9	126458	126492	+	(CTTT)n	Simple repeat
193	PBX1 intron 2	23.3	4.1	0.0	127180	127252	+	7SLRNA	srpRNA
194	PBX1 intron 2	22.4	5.6	0.0	127197	127303	-	SVA A	Retroposon/SVA
195	PBX1 intron 2	36.6	2.9	1.9	127472	127574	-	MIRc	SINE/MIR
196	PBX1 intron 2	10.3	0.3	0.0	127701	128001	-	AluSx	SINE/Alu
197	PBX1 intron 2	16.0	2.3	1.1	128468	128814	+	Tigger4b	DNA/TcMar-Tigger
198	PBX1 intron 2	32.8	0.0	0.0	129029	129086	+	L2a	LINE/L2
199	PBX1 intron 2	14.7	2.6	0.0	129402	129439	+	(ATATTGT)n	Simple repeat
200	PBX1 intron 2	33.5	8.8	1.3	129488	129635	-	MIRc	SINE/MIR
201	PBX1 intron 2	32.8	11.8	1.3	130814	131017	+	L2c	LINE/L2
202	PBX1 intron 2	32.2	0.0	0.0	131061	131175	+	L2b	LINE/L2
203	PBX1 intron 2	36.2	5.6	6.5	131502	131717	+	L2c	LINE/L2
204	PBX1 intron 2	20.4	8.7	0.9	131747	131954	+	Chap1a Mam	DNA/hAT-Charlie
205	PBX1 intron 2	33.7	13.5	2.7	132009	132416	+	L2c	LINE/L2
206	PBX1 intron 2	22.7	12.5	0.0	133174	133261	+	L3	LINE/CR1
207	PBX1 intron 2	21.7	0.0	0.0	133433	133464	+	(TTGACT)n	Simple repeat
208	PBX1 intron 2	31.7	5.3	2.3	133598	133768	+	MIRb	SINE/MIR
209	PBX1 intron 2	27.7	10.1	0.4	133771	133998	-	MIR	SINE/MIR
210	PBX1 intron 2	30.9	10.8	0.0	135248	135441	+	Tigger15a	DNA/TcMar-Tigger
211	PBX1 intron 2	26.0	10.7	0.4	135450	135673	-	MIR	SINE/MIR
212	PBX1 intron 2	25.0	10.7	9.9	135707	135986	+	Tigger15a	DNA/TcMar-Tigger
213	PBX1 intron 2	26.4	3.8	1.8	137491	137737	+	MIRc	SINE/MIR
214	PBX1 intron 2	26.7	3.3	0.0	138236	138295	+	AmnL2-1	LINE/L2
215	PBX1 intron 2	19.7	3.5	0.0	139715	140003	-	7SLRNA	srpRNA
216	PBX1 intron 2	3.6	13.8	0.0	140651	140679	+	GA-rich	Low complexity
217	PBX1 intron 2	15.8	0.9	0.5	141729	141944	-	MER20	DNA/hAT-Charlie
218	PBX1 intron 2	27.4	1.0	1.0	142995	143097	-	X6b DNA	DNA/TcMar-Tigger
219	PBX1 intron 2	31.7	1.2	0.0	143323	143404	-	AmnL2-1	LINE/L2
220	PBX1 intron 2	0.0	0.0	0.0	144084	144107	+	(GT)n	Simple repeat
221	PBX1 intron 2	0.0	0.0	0.0	144108	144145	+	(GA)n	Simple repeat
222	PBX1 intron 2	27.6	10.3	12.1	145003	145082	-	MIR	SINE/MIR
223	PBX1 intron 2	14.1	1.3	0.6	145083	145397	-	L1PA17	LINE/L1
224	PBX1 intron 2	27.6	10.3	12.1	145398	145559	-	MIR	SINE/MIR
225	PBX1 intron 2	28.8	6.3	1.5	145588	145714	-	L2b	LINE/L2
226	PBX1 intron 2	26.5	0.0	1.0	146736	146834	+	MIR	SINE/MIR
227	PBX1 intron 2	11.9	0.3	0.3	147088	147398	-	AluSx	SINE/Alu
228	PBX1 intron 2	7.7	0.0	0.0	147954	148250	-	AluY	SINE/Alu
229	PBX1 intron 2	0.0	0.0	0.0	148867	148883	+	(A)n	Simple repeat
230	PBX1 intron 2	12.5	0.0	3.7	150244	150271	+	(CCTT)n	Simple repeat
231	PBX1 intron 2	21.2	0.0	3.0	150921	150988	-	MIR3	SINE/MIR
232	PBX1 intron 2	25.0	1.8	0.0	151637	151692	+	MIR	SINE/MIR
233	PBX1 intron 2	21.4	1.6	28.8	151714	151842	+	L2d2	LINE/L2
234	PBX1 intron 2	29.5	4.8	0.0	152234	152338	+	L2a	LINE/L2
235	PBX1 intron 2	27.4	0.0	0.0	153263	153335	+	7SLRNA	srpRNA
236	PBX1 intron 2	24.1	6.4	0.9	153275	153374	-	SVA A	Retroposon/SVA

237	PBX1 intron 2	0.0	0.0	0.0	153375	153419	+	(AC)n	Simple repeat
238	PBX1 intron 2	27.6	9.2	1.9	154617	154820	-	MIRb	SINE/MIR
239	PBX1 intron 2	32.3	3.1	1.0	155742	155838	+	AmnSINE1	SINE/5S-Deu-L2
240	PBX1 intron 2	22.1	16.1	3.2	156195	156306	+	MER5B	DNA/hAT-Charlie
241	PBX1 intron 2	0.0	0.0	3.9	158113	158139	+	(GT)n	Simple repeat
242	PBX1 intron 2	25.8	3.4	0.4	158538	158687	-	L2a	LINE/L2
243	PBX1 intron 2	12.6	8.3	0.0	159291	159326	+	(TC)n	Simple repeat
244	PBX1 intron 2	29.2	3.0	3.0	159709	159775	-	MIR3	SINE/MIR
245	PBX1 intron 2	12.0	3.7	0.5	161064	161280	-	MER30	DNA/hAT-Charlie
246	PBX1 intron 2	0.0	0.0	0.0	161360	161398	+	(T)n	Simple repeat
247	PBX1 intron 2	22.2	31.5	0.6	162435	162561	-	MIRb	SINE/MIR
248	PBX1 intron 2	30.6	0.0	1.6	163980	164042	+	L2d2	LINE/L2
249	PBX1 intron 2	14.9	2.6	2.6	164718	164731	+	GA-rich	Low complexity
250	PBX1 intron 2	16.3	3.1	0.0	164732	165020	+	AluJb	SINE/Alu
251	PBX1 intron 2	14.9	2.6	2.6	165021	165044	+	GA-rich	Low complexity
252	PBX1 intron 2	14.0	3.1	0.0	166171	166202	+	(ATATAC)n	Simple repeat
253	PBX1 intron 2	22.1	2.3	0.0	166203	166208	+	(TATA)n	Simple repeat
254	PBX1 intron 2	27.4	2.6	4.6	166211	166474	-	MIR	SINE/MIR
255	PBX1 intron 2	26.8	2.9	4.5	166484	166551	+	(ATAA)n	Simple repeat
256	PBX1 intron 2	30.0	5.1	2.2	166552	166728	-	MIRb	SINE/MIR
257	PBX1 intron 2	26.5	25.3	0.0	166823	166905	+	MamSINE1	SINE/tRNA-RTE
258	PBX1 intron 2	13.2	0.0	0.0	167748	167772	+	(GT)n	Simple repeat
259	PBX1 intron 2	37.0	10.7	0.3	168988	169243	+	L2c	LINE/L2
260	PBX1 intron 2	6.3	1.3	0.0	169244	169322	+	MADE1	DNA/TcMar-Mariner
261	PBX1 intron 2	37.0	10.7	0.3	169323	169412	+	L2c	LINE/L2
262	PBX1 intron 2	31.0	10.0	3.3	169590	169819	+	L2c	LINE/L2
263	PBX1 intron 2	20.4	4.3	5.7	169923	170027	+	L2c	LINE/L2
264	PBX1 intron 2	31.4	0.9	2.8	170322	170429	+	UCON58	DNA
265	PBX1 intron 2	28.1	1.6	0.0	171241	171304	-	L2b	LINE/L2
266	PBX1 intron 2	28.4	0.0	0.0	172148	172186	+	GA-rich	Low complexity
267	PBX1 intron 2	19.1	0.0	8.9	173978	174026	+	A-rich	Low complexity
268	PBX1 intron 2	7.8	4.6	0.6	174061	174135	-	L1ME1	LINE/L1
269	PBX1 intron 2	11.4	1.3	0.0	174160	174467	-	AluSx	SINE/Alu
270	PBX1 intron 2	21.8	3.1	7.7	174468	174985	-	L1ME1	LINE/L1
271	PBX1 intron 2	21.0	8.9	2.8	174997	175265	-	AluJb	SINE/Alu
272	PBX1 intron 2	19.2	0.0	0.0	175268	175319	-	L1ME1	LINE/L1
273	PBX1 intron 2	0.0	0.0	0.0	176443	176479	+	(TG)n	Simple repeat
274	PBX1 intron 2	20.4	4.1	0.0	177062	177110	-	L2c	LINE/L2
275	PBX1 intron 2	23.8	0.0	1.6	177283	177346	-	L2b	LINE/L2
276	PBX1 intron 2	29.3	10.2	2.8	177350	177546	-	L2c	LINE/L2
277	PBX1 intron 2	0.0	0.0	0.0	178429	178464	+	(GT)n	Simple repeat
278	PBX1 intron 2	13.5	0.0	0.0	178950	179238	+	AluSx	SINE/Alu
279	PBX1 intron 2	30.1	2.5	5.9	180429	180710	-	MLT1J	LTR/ERVL-MaLR
280	PBX1 intron 2	22.4	0.0	0.0	181289	181346	+	L2a	LINE/L2
281	PBX1 intron 2	4.9	0.0	4.5	181564	181586	+	(GTG)n	Simple repeat
282	PBX1 intron 2	26.3	3.0	1.5	181677	181811	-	MIRb	SINE/MIR
283	PBX1 intron 2	11.5	0.0	10.1	183059	183396	-	AluSx	SINE/Alu
284	PBX1 intron 2	31.8	5.1	1.1	183682	183859	-	MIRb	SINE/MIR
285	PBX1 intron 2	30.2	1.0	2.1	183973	184068	+	(TA)n	Simple repeat
286	PBX1 intron 2	26.8	2.2	4.5	184796	184932	+	LTR90A	LTR
287	PBX1 intron 2	25.2	7.4	0.0	185214	185348	+	LTR90A	LTR
288	PBX1 intron 2	30.1	3.7	3.3	185775	186045	-	L1ME4c	LINE/L1
289	PBX1 intron 2	16.9	0.0	0.0	186354	186387	+	(GGAT)n	Simple repeat
290	PBX1 intron 2	18.0	1.6	2.5	186565	186809	+	AluJb	SINE/Alu
291	PBX1 intron 2	0.0	0.0	0.0	186835	186872	+	(GT)n	Simple repeat
292	PBX1 intron 2	18.8	7.2	5.9	187647	187722	+	Charlie1b	DNA/hAT-Charlie
293	PBX1 intron 2	8.4	0.3	0.0	187723	188021	+	AluSx	SINE/Alu
294	PBX1 intron 2	18.8	7.2	5.9	188022	188270	+	Charlie1b	DNA/hAT-Charlie
295	PBX1 intron 2	10.7	0.3	0.3	188271	188570	-	AluSx	SINE/Alu
296	PBX1 intron 2	18.8	7.2	5.9	188571	188729	+	Charlie1b	DNA/hAT-Charlie

297	PBX1 intron 2	0.0	0.0	0.0	188896	188935	+	(TG)n	Simple repeat
298	PBX1 intron 2	28.2	0.0	5.2	190093	190153	+	(TTGTTT)n	Simple repeat
299	PBX1 intron 2	22.0	0.0	0.0	191348	191392	+	(GTGTGT)n	Simple repeat
300	PBX1 intron 2	11.2	4.8	0.0	191887	192137	-	AluSx	SINE/Alu
301	PBX1 intron 2	7.4	0.0	0.0	192688	192716	+	(A)n	Simple repeat
302	PBX1 intron 2	5.5	0.0	0.0	194390	194408	+	(TAT)n	Simple repeat
303	PBX1 intron 2	21.3	0.0	0.0	195514	195574	+	L2b	LINE/L2
304	PBX1 intron 2	29.9	6.8	2.6	195632	195851	+	L1ME3A	LINE/L1
305	PBX1 intron 2	20.1	1.7	0.8	197053	197172	+	(CCTT)n	Simple repeat
306	PBX1 intron 2	7.0	10.0	0.0	197249	197279	+	A-rich	Low complexity
307	PBX1 intron 2	3.4	0.3	0.0	198137	198431	-	AluY	SINE/Alu
308	PBX1 intron 2	28.7	7.0	3.2	199618	199787	-	MIRc	SINE/MIR
309	PBX1 intron 2	29.8	3.5	4.1	199880	200222	-	MLT1J	LTR/ERVL-MaLR
310	PBX1 intron 2	25.6	8.6	5.8	201122	201341	-	UCON64	Unknown
311	PBX1 intron 2	29.2	0.8	2.4	202233	202485	-	MIRb	SINE/MIR
312	PBX1 intron 2	29.5	1.1	0.0	202620	202714	-	L2d2	LINE/L2
313	PBX1 intron 2	24.3	1.4	0.0	203233	203302	-	MIRc	SINE/MIR
314	PBX1 intron 2	9.4	3.1	1.0	203387	203702	-	AluSx	SINE/Alu
315	PBX1 intron 2	11.0	6.0	0.0	203703	203726	+	(TTTTC)n	Simple repeat
316	PBX1 intron 2	23.7	2.4	0.0	203894	204062	-	MER45A	DNA/hAT-Tip100
317	PBX1 intron 2	5.3	0.0	0.0	204339	204358	+	(A)n	Simple repeat
318	PBX1 intron 2	28.2	14.2	2.0	204896	205029	-	MIRb	SINE/MIR
319	PBX1 intron 2	13.7	0.3	0.7	205477	205771	+	AluSx	SINE/Alu
320	PBX1 intron 2	30.7	8.0	0.0	206174	206261	+	MIRc	SINE/MIR
321	PBX1 intron 2	27.0	13.5	0.0	206954	207042	-	MIRc	SINE/MIR
322	PBX1 intron 2	0.0	0.0	0.0	209933	209962	+	(CA)n	Simple repeat
323	PBX1 intron 2	27.4	4.1	5.5	212684	212830	+	L2c	LINE/L2
324	PBX1 intron 2	0.0	0.0	0.0	213589	213624	+	(TG)n	Simple repeat
325	PBX1 intron 2	0.0	0.0	0.0	213632	213668	+	(GA)n	Simple repeat
326	PBX1 intron 2	14.0	4.5	0.0	213843	214128	+	AluSx	SINE/Alu
327	PBX1 intron 2	15.2	2.4	0.0	215051	215345	+	AluJb	SINE/Alu
328	PBX1 intron 2	20.8	10.2	2.2	215615	215731	+	MIR	SINE/MIR
329	PBX1 intron 2	3.6	0.0	0.0	215732	215814	-	AluY	SINE/Alu
330	PBX1 intron 2	24.8	9.2	3.4	217711	217906	-	MamSINE1	SINE/tRNA-RTE
331	PBX1 intron 2	10.6	2.1	0.0	218725	218771	-	MLT1J	LTR/ERVL-MaLR
332	PBX1 intron 2	26.0	1.8	0.0	220064	220118	+	(GTGT)n	Simple repeat
333	PBX1 intron 2	15.8	0.0	3.5	221230	221259	+	(ACAA)n	Simple repeat
334	PBX1 intron 2	10.3	0.0	2.6	221411	221450	+	MIRb	SINE/MIR
335	PBX1 intron 2	30.4	6.2	7.6	223035	223194	-	L2d2	LINE/L2
336	PBX1 intron 2	0.0	0.0	0.0	223371	223410	+	(TG)n	Simple repeat
337	PBX1 intron 2	29.2	0.0	0.0	225049	225090	+	(TTT)n	Simple repeat
338	PBX1 intron 2	7.9	3.7	0.0	225454	225480	+	(CTTTT)n	Simple repeat
339	PBX1 intron 2	32.3	1.3	0.0	227184	227262	+	(CTTC)n	Simple repeat
340	PBX1 intron 2	16.5	15.4	9.0	228473	228532	-	L1MB3	LINE/L1
341	PBX1 intron 2	10.0	0.4	2.4	228533	228789	-	AluY	SINE/Alu
342	PBX1 intron 2	16.5	15.4	9.0	228790	228981	-	L1MB3	LINE/L1

Table S5: Repeats in *TCF3* intron 16

#	query	%div.	%del.	%ins.	begin	end	orientation	repeat	class/family
1	TCF3 intron 16	28.6	14.8	0.8	337	1058	-	L2a	LINE/L2
2	TCF3 intron 16	24.7	5.1	0.0	1182	1359	+	MER20	DNA/hAT-Charlie
3	TCF3 intron 16	29.5	2.3	3.8	1979	2193	-	L1M5	LINE/L1
4	TCF3 intron 16	24.8	4.7	2.5	2312	2543	-	AluJb	SINE/Alu
5	TCF3 intron 16	20.3	7.2	0.0	2544	2612	+	U6	snRNA
6	TCF3 intron 16	10.0	0.0	0.0	2616	2904	-	AluY	SINE/Alu

Table S6: Potential cryptic recombination signal sites in *PBX1* intron 2

#	query	begin	end	type	sequence	orientation
1	PBX1 intron 2	434	472	RSS23	cacaaagcagcagactagaggttctcaacagagagtgtt	+
2	PBX1 intron 2	2113	2140	RSS12	cacacacacacacacacacacacacaca	+
3	PBX1 intron 2	2115	2142	RSS12	cacacacacacacacacacacacacaca	+
4	PBX1 intron 2	2117	2155	RSS23	cacacacacacacacacacacacaagtcttggattaa	+
5	PBX1 intron 2	2119	2146	RSS12	cacacacacacacacacacacaagtc	+
6	PBX1 intron 2	2123	2161	RSS23	cacacacacacacacacacaagtcttggattaagagggc	+
7	PBX1 intron 2	2129	2167	RSS23	cacacacacacacaagtcttggattaagagggcacccct	+
8	PBX1 intron 2	3370	3397	RSS12	cacaggaataaaaaacatcaggaaatgt	-
9	PBX1 intron 2	3637	3675	RSS23	cacagagagaaaggaaagagagagaacaaaagggtgtt	-
10	PBX1 intron 2	4497	4524	RSS12	cacagacactcctattgttagaaaaaaa	-
11	PBX1 intron 2	4846	4884	RSS23	cacataatttaaattggaagacattttcatttgtaaagc	+
12	PBX1 intron 2	4938	4976	RSS23	catagtatcatccatctaaaagagatttgaaaccaaatt	-
13	PBX1 intron 2	5168	5206	RSS23	cacaccattgcaggtaaagggcaatggaaccaaaattat	-
14	PBX1 intron 2	5247	5285	RSS23	cacaggaactcaaagcacttcacaaacaactcatgaatt	-
15	PBX1 intron 2	5256	5283	RSS12	caggaactcaaagcacttcacaaacaac	-
16	PBX1 intron 2	5258	5285	RSS12	cacaggaactcaaagcacttcacaaaca	-
17	PBX1 intron 2	5509	5547	RSS23	cagagtgatccccagaggggaaaaaggtgcaggaatag	+
18	PBX1 intron 2	6733	6771	RSS23	cacaatgcctccagttgctgacgctaagaaaacaccatc	-
19	PBX1 intron 2	7054	7092	RSS23	caccccctgacaggccccagtgtgtgatgttcccctccc	+
20	PBX1 intron 2	7066	7104	RSS23	cacatggacacagggaggggaacatcacacactggggcc	-
21	PBX1 intron 2	7193	7220	RSS12	cagcttcatccatgtccctgcaaagaac	+
22	PBX1 intron 2	7234	7261	RSS12	caccatggaatactatggagccataaaa	-
23	PBX1 intron 2	7471	7498	RSS12	cactgtcttccacaatggttgaataatt	+
24	PBX1 intron 2	7501	7528	RSS12	cactgtcaccaacagtgtaaaagaattc	+
25	PBX1 intron 2	7705	7732	RSS12	cagtgggtgaaggatatgaacagacact	-
26	PBX1 intron 2	8855	8893	RSS23	caaagtgctggagttacaggtgtgaggcaccgcgcccag	+
27	PBX1 intron 2	9501	9528	RSS12	cagagtgttgaccacctttctcatcagt	+
28	PBX1 intron 2	9597	9635	RSS23	cactgacaaccatttaacataaaagcagggtcaggaagc	-
29	PBX1 intron 2	9610	9637	RSS12	cacactgacaaccatttaacataaaagc	-
30	PBX1 intron 2	9683	9721	RSS23	cacagaactctagaagtaggggaggggggtgtgaaagtg	-
31	PBX1 intron 2	10884	10911	RSS12	cagtctattctagggaacacttaagatt	-
32	PBX1 intron 2	11391	11429	RSS23	caaagtgctgggattataggtgtgagccaccacacccag	-
33	PBX1 intron 2	11747	11785	RSS23	catagcatgaaaacaacatatgcattccatcacaaagtc	-
34	PBX1 intron 2	12023	12061	RSS23	cactttcttagggaaactcaaacatctttctaaatatcc	-
35	PBX1 intron 2	12089	12116	RSS12	caacctgtctcaaagcaagacaaatata	-
36	PBX1 intron 2	14284	14311	RSS12	cactagaatacacttaagaataataatc	-
37	PBX1 intron 2	14832	14859	RSS12	cacaaacaatctttgcatacttataccc	-
38	PBX1 intron 2	15795	15833	RSS23	cacagaggtaaatttcacacttcctctcagtactccttc	-
39	PBX1 intron 2	16134	16161	RSS12	cacagtgagaaagacatcacacggaata	-
40	PBX1 intron 2	16427	16454	RSS12	catcctggaatgaaactgaacagagaat	+
41	PBX1 intron 2	17527	17565	RSS23	cagagtgctgggattataggtatgagccaccgcacctgg	+
42	PBX1 intron 2	18184	18222	RSS23	caaaaggggggctgggtgcggtggctcatgcctgtaatcc	-
43	PBX1 intron 2	18241	18279	RSS23	cactgcaatttggaaggtagatcttccctcctcaaaact	-
44	PBX1 intron 2	18552	18590	RSS23	cacagccataaggaaagtaacacaagaaaatgtaaacac	-
45	PBX1 intron 2	18563	18590	RSS12	cacagccataaggaaagtaacacaagaa	-
46	PBX1 intron 2	18827	18854	RSS12	cacaataagaaaggatcttatggatacc	-
47	PBX1 intron 2	19452	19479	RSS12	cattgccctcaacgacatgataaaaaat	+
48	PBX1 intron 2	20498	20536	RSS23	cagagtgagaaatataagataggaacttgtcctcaaatt	+
49	PBX1 intron 2	21832	21870	RSS23	cacattggaaggggttggggaaggagtgaggagtgtgcc	-
50	PBX1 intron 2	22222	22249	RSS12	caatgcctgactacccccctcaaagact	-
51	PBX1 intron 2	23080	23118	RSS23	cacatgggctcagatgtttccattccaccttggaaatga	-
52	PBX1 intron 2	23093	23131	RSS23	cataatgctgctccacatgggctcagatgtttccattcc	-
53	PBX1 intron 2	23394	23432	RSS23	cacagattggcaggcatgtggagggctgatttagaacat	+
54	PBX1 intron 2	23748	23786	RSS23	cacacacacacaaacaacaccctatgctgctcaatgtca	-
55	PBX1 intron 2	23765	23792	RSS12	cagggacacacacacacaaacaacaccc	-
56	PBX1 intron 2	24197	24224	RSS12	cacaaagatcatcagctaacaacaaaga	-

57	PBX1 intron 2	24428	24466	RSS23	cacacacagacacagagttctcacttgcagcagtccggc	+
58	PBX1 intron 2	24432	24459	RSS12	cacagacacagagttctcacttgcagca	+
59	PBX1 intron 2	24595	24622	RSS12	cacagtcactgaagtcccatttttatat	+
60	PBX1 intron 2	25113	25151	RSS23	cacacagagatgaggaaaagagaaaagagaaatacact	+
61	PBX1 intron 2	25115	25153	RSS23	cacagagatgaggaaaagagaaaagagaaatacactcc	+
62	PBX1 intron 2	25177	25215	RSS23	cacacttcaccatatgcttcgaaactaaaaaaaaaaaa	-
63	PBX1 intron 2	25742	25769	RSS12	caaagtggcgcagaccttttcctgactc	+
64	PBX1 intron 2	25758	25796	RSS23	cactgagtgatgaggacaaagggtgaggagtcaggaaaa	-
65	PBX1 intron 2	26366	26404	RSS23	catggtgacaggtggataatatctcctccaccctaaata	-
66	PBX1 intron 2	26505	26543	RSS23	caccacaacaggaacagaaatctaattcaacttcaatca	-
67	PBX1 intron 2	27104	27142	RSS23	cacagagcagcttgtttacctgaagcacagggggggcggc	-
68	PBX1 intron 2	27196	27234	RSS23	cacactgttgtcctcctcattaaggctcaccaaaaacgg	-
69	PBX1 intron 2	27960	27998	RSS23	caaagtgctgggattacaggcgtcagccaccgctcctgg	+
70	PBX1 intron 2	28579	28606	RSS12	cacagaaggccccaactttgcaaatgcc	-
71	PBX1 intron 2	28827	28865	RSS23	cacagacttgaacacaacttgatttgaacaaaggccctc	-
72	PBX1 intron 2	28913	28951	RSS23	cacagggctcatagtttgagtgtgagagtctggatgagg	+
73	PBX1 intron 2	29227	29254	RSS12	cagcaccctcagactctgctcagacact	-
74	PBX1 intron 2	29248	29275	RSS12	caaagcctctgtggccagggccagcacc	-
75	PBX1 intron 2	29483	29521	RSS23	caccaggctcccacgcgctggccttgctgaggataaaga	+
76	PBX1 intron 2	29758	29796	RSS23	cacacacctgtaatgccagttacttgggaggctgaggca	-
77	PBX1 intron 2	29874	29912	RSS23	cactttgcaaggccgaggcaggcagatcacttgagccca	-
78	PBX1 intron 2	29997	30024	RSS12	caccaggagtcaaaatccaacaaaatga	-
79	PBX1 intron 2	30092	30130	RSS23	cacagtatttctaacctttggggcatagattaattct	-
80	PBX1 intron 2	30253	30280	RSS12	cacatttttatagatgaaaacaaaacag	-
81	PBX1 intron 2	30393	30431	RSS23	cactgggttgaaaacacaccatgtacttcctttaaaata	+
82	PBX1 intron 2	30919	30957	RSS23	cagaatgctaaagctgcaacagagacttgggagatcatt	+
83	PBX1 intron 2	31229	31267	RSS23	caaagtgctgggattacaggcatgagccactgtgcccgg	+
84	PBX1 intron 2	31513	31540	RSS12	cacagtggctagtggcaaccttactaga	+
85	PBX1 intron 2	31583	31610	RSS12	cactgaggcccagaactgtgattaacta	+
86	PBX1 intron 2	32051	32089	RSS23	cacagccaacttgccaccgtgggaccactactgaaaact	-
87	PBX1 intron 2	34142	34169	RSS12	cacccccaaatagctccatttcaatacc	-
88	PBX1 intron 2	34735	34762	RSS12	caccatgtgatgaactccaccatgttac	-
89	PBX1 intron 2	34804	34831	RSS12	caaagcaattcccacagtaacaaaccca	+
90	PBX1 intron 2	34816	34854	RSS23	cacagtaacaaacccactcctaggatagcaatgttaatc	+
91	PBX1 intron 2	35476	35503	RSS12	cattgtgtcatacctcggagcgtatagc	+
92	PBX1 intron 2	37313	37351	RSS23	cactgtgttaaacaggcatggggctgcctcaggaatcaa	-
93	PBX1 intron 2	37499	37537	RSS23	cacaccgatcaagattgatccagccactgaagtaatcca	+
94	PBX1 intron 2	37646	37684	RSS23	caaagtgctgggattacaggcatgagccactgcgcccag	-
95	PBX1 intron 2	38439	38466	RSS12	cacagtatccaactgcagcatttaaatg	-
96	PBX1 intron 2	38625	38663	RSS23	cacagagctgaatccccactcctagggtgcatggctgag	-
97	PBX1 intron 2	39242	39269	RSS12	cacagactcatacagcctccacagacac	+
98	PBX1 intron 2	39261	39299	RSS23	cacagacacaaagcttctgtgtatcctacatgtacctgc	+
99	PBX1 intron 2	39808	39835	RSS12	cattctgactctgttcctcatggttact	-
100	PBX1 intron 2	39896	39934	RSS23	cacaaagagataaaaggaaaagcagaaaaaaataaaatg	-
101	PBX1 intron 2	39905	39932	RSS12	caaagagataaaaggaaaagcagaaaaa	-
102	PBX1 intron 2	40991	41018	RSS12	catagtgtggtcaagaacaacttacata	-
103	PBX1 intron 2	41063	41101	RSS23	cacagtgttcccattactctaagggttacaatctgaggt	+
104	PBX1 intron 2	41445	41472	RSS12	cacaaaagaatgactcagttctacacca	-
105	PBX1 intron 2	41506	41533	RSS12	cacagtatttcataaatgaaaaaaaaaa	-
106	PBX1 intron 2	41510	41548	RSS23	cactttgtctaaaagcacagtatttcataaatgaaaaaa	-
107	PBX1 intron 2	44620	44658	RSS23	cacagagctcacagacataggaaaacctgatagaaaata	-
108	PBX1 intron 2	44887	44925	RSS23	cacagtacttggcacatgctgggcactcagtaagtatct	-
109	PBX1 intron 2	46021	46048	RSS12	caccctgcttcagcatgttaggcacatt	+
110	PBX1 intron 2	46981	47019	RSS23	cacagagccctggaaccagccccttcttgcttcaaaggt	-
111	PBX1 intron 2	47317	47344	RSS12	cacagggacacagtacagaaccgatcgc	-
112	PBX1 intron 2	47888	47926	RSS23	cacagaaatgtgaaaaaggcccaaagttagagagaaaaa	-
113	PBX1 intron 2	48000	48038	RSS23	cactgcaagctccgcctcccgggttcatgccattcttct	+
114	PBX1 intron 2	48186	48224	RSS23	caaagtgctgggattacaggcgtgagccaccgcgcccgg	+
115	PBX1 intron 2	48195	48233	RSS23	cacagaaggccgggcgcggtggctcacgcctgtaatccc	-
116	PBX1 intron 2	49378	49416	RSS23	cactgccctaggcggaggaagagatgcagaatatgatgg	+

117	PBX1 intron 2	50194	50232	RSS23	cacaatgctaggggggctcacaggagagcctggagaaag	-
118	PBX1 intron 2	50264	50291	RSS12	cacatatacacagatcataaccgcaaca	-
119	PBX1 intron 2	50687	50725	RSS23	cacagaaataccagaggaaggtcatcctgggagttgatg	-
120	PBX1 intron 2	50960	50998	RSS23	caaagtgtggttaagtgactataccgaggacacagacct	+
121	PBX1 intron 2	51691	51729	RSS23	cactttgggaggccgaagtgggcagatcacctgaggtca	-
122	PBX1 intron 2	51750	51788	RSS23	cactgtgcctggcctcagttgaactctttaagattattc	+
123	PBX1 intron 2	52163	52190	RSS12	cacagtataaaatcaggaatattacacc	-
124	PBX1 intron 2	52614	52652	RSS23	cacaataccctggcctccccagactcctggctcttaacc	-
125	PBX1 intron 2	52705	52743	RSS23	cacatagacatatcctgaatttttcatctctggagaccc	-
126	PBX1 intron 2	53495	53522	RSS12	catagtgctaaacccattgacatagatc	+
127	PBX1 intron 2	55789	55827	RSS23	cagggagagaaacaaaagaaggaaactcacaggaaacca	+
128	PBX1 intron 2	55839	55866	RSS12	cactgccaggcacaccccttttgaaacc	+
129	PBX1 intron 2	56070	56097	RSS12	cactttgttggagctatttacaatatct	+
130	PBX1 intron 2	56694	56721	RSS12	caacatagtgagaccctgtttctacaaa	+
131	PBX1 intron 2	57182	57220	RSS23	cacagggtggtgaggtggaacaaaaaagaaaaaagattg	-
132	PBX1 intron 2	57193	57220	RSS12	cacagggtggtgaggtggaacaaaaaag	-
133	PBX1 intron 2	57631	57669	RSS23	caaagtgctggggttacaggcgtgagccactgcgcccgg	+
134	PBX1 intron 2	57738	57776	RSS23	cacagtcttggctcactgcaacctccgcctcccggattc	+
135	PBX1 intron 2	57981	58019	RSS23	cagacagacagaaagagaaggaagactgttaagaaaata	-
136	PBX1 intron 2	58192	58230	RSS23	cagagccaccaagctaaatcagcccctgaaggagaacct	-
137	PBX1 intron 2	58537	58564	RSS12	caatttgacttccatcttccttaagaat	+
138	PBX1 intron 2	58959	58986	RSS12	cacaacagtgcggcccagagagaaatgt	-
139	PBX1 intron 2	59019	59057	RSS23	cacagagattctaggctaacctctgtggacttgacattt	-
140	PBX1 intron 2	59411	59449	RSS23	cacaccagcaaagtggttgtgggttgtgatttcaaacag	-
141	PBX1 intron 2	59458	59496	RSS23	cacacacacacactgacacatgcttgctatacatacata	+
142	PBX1 intron 2	59466	59493	RSS12	cacactgacacatgcttgctatacatac	+
143	PBX1 intron 2	59761	59799	RSS23	caaagtgctaggattacaggcatgagccaacacgcctgg	+
144	PBX1 intron 2	60303	60341	RSS23	cagagagacaaaggcaagcacagagaggagatgcaaggc	-
145	PBX1 intron 2	60314	60341	RSS12	cagagagacaaaggcaagcacagagagg	-
146	PBX1 intron 2	60778	60805	RSS12	cactgtcctaaaaccacagggctaactg	+
147	PBX1 intron 2	60778	60816	RSS23	cactgtcctaaaaccacagggctaactgtatctgaagaa	+
148	PBX1 intron 2	61300	61338	RSS23	catggacacacatgcagataaatatacctactaaaattc	+
149	PBX1 intron 2	61524	61551	RSS12	cacagaggttgggagcttctgtctagcc	+
150	PBX1 intron 2	62371	62409	RSS23	cacacacactcacatacacaaagatgaaagatcttatga	-
151	PBX1 intron 2	62550	62577	RSS12	caccctccttctgcctttctgtaaaatc	+
152	PBX1 intron 2	62761	62788	RSS12	cacctttgccttctccccacccaaaact	-
153	PBX1 intron 2	64050	64077	RSS12	cacatcggtaccaacacactcaaaaatc	-
154	PBX1 intron 2	64187	64225	RSS23	cacgatgctgcgggtccattctcaaatgccaccacctct	-
155	PBX1 intron 2	64312	64339	RSS12	cagcctagtctagcttccccaaaacatc	-
156	PBX1 intron 2	64712	64750	RSS23	cactgtgctaggaacagatgtccccaaaaagcaattaac	-
157	PBX1 intron 2	65548	65575	RSS12	cagcacccccttacaccgtcacatatttt	+
158	PBX1 intron 2	65943	65970	RSS12	cacaaagctccatatttaactcaaaatg	-
159	PBX1 intron 2	67410	67448	RSS23	caatgcaactcagaggaaagagtatgggctttgaaatca	-
160	PBX1 intron 2	67998	68025	RSS12	caaagtgctttctgaacaaacttaaatt	+
161	PBX1 intron 2	69270	69308	RSS23	cacagtgttgaatccacggtgggcattctgcatgttcta	-
162	PBX1 intron 2	69652	69679	RSS12	cactctaggttttcttatctataaaaca	-
163	PBX1 intron 2	70098	70136	RSS23	caaagtgctaggattacaggcgtgagccacagcacgcag	+
164	PBX1 intron 2	70311	70349	RSS23	cactacattacacttttccttgtatagtacaggaaatct	-
165	PBX1 intron 2	70428	70466	RSS23	cacattatttaacctctctttgcttcagtttcctaatct	+
166	PBX1 intron 2	70528	70566	RSS23	cagaaagcaattaaaacagtgcctggtgcatgttaagcc	+
167	PBX1 intron 2	70723	70761	RSS23	cacagccgtggatgaacagtttcttccttgtgagccagc	+
168	PBX1 intron 2	71130	71157	RSS12	cactctcttttcaaagtgttcatgaaat	-
169	PBX1 intron 2	71243	71281	RSS23	cacaatgctctttgggtttcgtttctggaatatgaattg	+
170	PBX1 intron 2	71678	71705	RSS12	cagaatattcgcagccttagagttaact	-
171	PBX1 intron 2	72108	72135	RSS12	cacaaattaatatactctacttaaacca	+
172	PBX1 intron 2	72299	72326	RSS12	caccctccatccagcctcgccatatatc	+
173	PBX1 intron 2	73032	73059	RSS12	caccttgttccaagctgaaataagatgc	-
174	PBX1 intron 2	74783	74821	RSS23	cacacacagccatagcagcagtacgagtgcccttaactg	-
175	PBX1 intron 2	75159	75186	RSS12	cagaggggcctagcacttttcaaagatt	-
176	PBX1 intron 2	75264	75302	RSS23	cacatacactcacacacactcacaccatgggtgaaaacg	+

177	PBX1 intron 2	75420	75447	RSS12	caccgtcacacatgacattcttgaatga	+
178	PBX1 intron 2	75796	75834	RSS23	cactctgtaaaaccgatattgctatcccttacagacaag	-
179	PBX1 intron 2	77506	77544	RSS23	caaagtgctaggattacaggcatgagccactgcagcggc	+
180	PBX1 intron 2	77575	77613	RSS23	cacagtattctgggcaaaccagatgttaatagcttgacc	+
181	PBX1 intron 2	78164	78202	RSS23	cacagagatgaaaccccgtggaactagggcacgggaaga	-
182	PBX1 intron 2	78408	78435	RSS12	cacagtattctctgacttagaataaaat	-
183	PBX1 intron 2	78788	78826	RSS23	cacaatggaaataaacagtactagagatggggcttatcc	-
184	PBX1 intron 2	79063	79101	RSS23	caccaacatgatttctccttttggtctcagaccaggaca	+
185	PBX1 intron 2	79270	79308	RSS23	cacctaaccaaataaataaattcatactgaacacttacc	-
186	PBX1 intron 2	79469	79507	RSS23	cacagtgacaccagtccaagcagagagcagagactgctt	-
187	PBX1 intron 2	79480	79507	RSS12	cacagtgacaccagtccaagcagagagc	-
188	PBX1 intron 2	79501	79539	RSS23	cactgtgatcattctaaggtaacgagccaccttttattc	+
189	PBX1 intron 2	80139	80166	RSS12	cactttctagccagtccacacaagaacc	+
190	PBX1 intron 2	80528	80566	RSS23	cacagtgattttgtacaagggctttggagtcaggtagac	-
191	PBX1 intron 2	81625	81663	RSS23	caaagtcagatttgaaaaacggctttattaagagaaaga	-
192	PBX1 intron 2	82382	82420	RSS23	cacagatatatacctcactcccgattaatgtgcaaattt	+
193	PBX1 intron 2	82616	82654	RSS23	cacagtcacacagtctgtgtcttctgaggcttggatcca	-
194	PBX1 intron 2	82769	82807	RSS23	cacagagagaaaaataaaatcccttcagattcgcatctt	-
195	PBX1 intron 2	83785	83823	RSS23	cactgggtttcaaaggttacttaaaagcagactctttcc	-
196	PBX1 intron 2	83796	83823	RSS12	cactgggtttcaaaggttacttaaaagc	-
197	PBX1 intron 2	84355	84382	RSS12	cagagtggttttcaaccctggctgaaca	+
198	PBX1 intron 2	84763	84801	RSS23	caaagtgctgggattgcaggcgtgagccaccgtgcccag	+
199	PBX1 intron 2	84810	84837	RSS12	cacaaccccagtgcataacacaaaagct	-
200	PBX1 intron 2	85661	85688	RSS12	cagcctaagtcacatcaatcacaaaact	-
201	PBX1 intron 2	85713	85751	RSS23	cacacctctgtggggttactttgtgttcattctggacct	+
202	PBX1 intron 2	86222	86249	RSS12	cacataggaacaactaacaccagaaaac	-
203	PBX1 intron 2	86783	86821	RSS23	cacaatgtgaggcccggggatgggatctgtagcactaca	-
204	PBX1 intron 2	86949	86976	RSS12	cagtgccctgcagcctgggcaaaagagc	-
205	PBX1 intron 2	87387	87425	RSS23	cactgtgcaaagtccttaatgtgcacacctagaggagca	-
206	PBX1 intron 2	87619	87646	RSS12	caacatcattccccccattttcaaccac	-
207	PBX1 intron 2	88140	88178	RSS23	cacagagagcgggccctgcagtgttgagcctaatgaatt	+
208	PBX1 intron 2	88279	88317	RSS23	caggggaagagggaggtgcagagaacacataaacaaccc	+
209	PBX1 intron 2	88394	88432	RSS23	caccatgcttaaagtggatttggtcgcaggaggaaagtc	-
210	PBX1 intron 2	88598	88636	RSS23	cacacacacacacacaccctccctccataagctctgact	-
211	PBX1 intron 2	88602	88640	RSS23	cacacacacacacacacacaccctccctccataagctct	-
212	PBX1 intron 2	88604	88642	RSS23	cacacacacacacacacacacaccctccctccataagct	-
213	PBX1 intron 2	88605	88632	RSS12	cacacacacacaccctccctccataagc	-
214	PBX1 intron 2	88614	88652	RSS23	cacacacacacacacacacacacacacacacaccctccc	-
215	PBX1 intron 2	88616	88654	RSS23	cacacacacacacacacacacacacacacacacaccctc	-
216	PBX1 intron 2	88618	88656	RSS23	cacacacacacacacacacacacacacacacacacaccc	-
217	PBX1 intron 2	88619	88646	RSS12	cacacacacacacacacacacacacacc	-
218	PBX1 intron 2	88621	88648	RSS12	cacacacacacacacacacacacacaca	-
219	PBX1 intron 2	88623	88650	RSS12	cacacacacacacacacacacacacaca	-
220	PBX1 intron 2	88625	88652	RSS12	cacacacacacacacacacacacacaca	-
221	PBX1 intron 2	88627	88654	RSS12	cacacacacacacacacacacacacaca	-
222	PBX1 intron 2	88629	88656	RSS12	cacacacacacacacacacacacacaca	-
223	PBX1 intron 2	88631	88658	RSS12	cacacacacacacacacacacacacaca	-
224	PBX1 intron 2	90802	90840	RSS23	cacagtgcattgggggtagggtgtctagctctaatattt	+
225	PBX1 intron 2	91557	91595	RSS23	cacattactccccaatcacaagctcctgatggcagaggc	-
226	PBX1 intron 2	92264	92291	RSS12	cacactgtcttgactgtctcagtaaacc	-
227	PBX1 intron 2	92320	92347	RSS12	cacaaacagtaagtcaatcacaagaaaa	-
228	PBX1 intron 2	92489	92527	RSS23	catagtagtttcactgcatattgatgccatgtaaaatcc	-
229	PBX1 intron 2	92832	92870	RSS23	cactgagtagccatatcgatagatcctaacaattattca	-
230	PBX1 intron 2	94197	94224	RSS12	cattctagcagatgtatttcttgatacc	+
231	PBX1 intron 2	94464	94502	RSS23	cacagatcagggtgagcaaatgaaaggcaaatctaactc	-
232	PBX1 intron 2	94504	94542	RSS23	caggttgcatgggcttaaactgtagtggtcaataaagcc	-
233	PBX1 intron 2	94521	94559	RSS23	cagtttaagcccatgcaacctgcatgctgtgcaagaaag	+
234	PBX1 intron 2	95149	95176	RSS12	cacagccattatctgtattaatagatct	-
235	PBX1 intron 2	95211	95249	RSS23	cacactcggggatatcaagctgcaatccagagaaaagc	-
236	PBX1 intron 2	95480	95518	RSS23	cacagggtaacagataacatatgtaaagtggtaataaca	-

237	PBX1 intron 2	95491	95518	RSS12	cacagggtaacagataacatatgtaaag	-
238	PBX1 intron 2	97289	97316	RSS12	cactaagtgtgacctctagacaaaacca	-
239	PBX1 intron 2	97811	97849	RSS23	cacagtcaaataaagaagagttggtagacagagaaaaac	-
240	PBX1 intron 2	98078	98105	RSS12	cagtgaaattgctggtgatccctagacc	-
241	PBX1 intron 2	98375	98413	RSS23	caaagtgctgggattacaggcgtgagccactgtgcccag	+
242	PBX1 intron 2	98445	98472	RSS12	cacattaactaataccaaaaataaaatt	-
243	PBX1 intron 2	98767	98805	RSS23	cactttgggaggcctaggtgggcaggtcacctgaggtca	+
244	PBX1 intron 2	99113	99140	RSS12	cacagcttcaaggtattctgcaaaaatc	-
245	PBX1 intron 2	99526	99564	RSS23	caaagttctgcatattaaccatcactacaagaaaaaaaa	-
246	PBX1 intron 2	100082	100109	RSS12	catagggagaagcaatcctactaagata	-
247	PBX1 intron 2	100997	101035	RSS23	cacagacatatgactagagggacaccaactaaaaaactc	-
248	PBX1 intron 2	101151	101178	RSS12	cacagtcaggtgtgaccaaaccaaaact	+
249	PBX1 intron 2	101263	101290	RSS12	cagaataatgcagaatgatgccaggact	-
250	PBX1 intron 2	101319	101357	RSS23	cacactgctgataaagacatacccaagaccggacaaatt	+
251	PBX1 intron 2	101746	101773	RSS12	cacagaatttattacagaattcaaactc	-
252	PBX1 intron 2	102047	102085	RSS23	cactgtaacctctacctcccaggtttaagtgatcctccc	+
253	PBX1 intron 2	102235	102273	RSS23	caaagtgctgggattacaggtgtgagccactgaacccag	+
254	PBX1 intron 2	102345	102372	RSS12	catagggtagctgggtgctacaaacaca	-
255	PBX1 intron 2	103214	103241	RSS12	cagaatatttgctccatcagccagaatc	-
256	PBX1 intron 2	103621	103659	RSS23	cactctgtgacaaagagggttccataacatctctaagcc	-
257	PBX1 intron 2	104077	104115	RSS23	cacacacacacacatattatacatatgttatataaaata	-
258	PBX1 intron 2	104094	104121	RSS12	cacacacacacacacacacatattatac	-
259	PBX1 intron 2	104299	104326	RSS12	cacagtagatcattggcattcaaagatt	+
260	PBX1 intron 2	104853	104891	RSS23	cagagtgctgaaaagacaagttcaggaccatgtagccaa	-
261	PBX1 intron 2	105133	105171	RSS23	cacctccaccatgacttaggatcacctgagagagaaata	+
262	PBX1 intron 2	105238	105265	RSS12	cagggagcctccgagccctgccaaaatc	+
263	PBX1 intron 2	105265	105292	RSS12	cacttggccacagccccaagataatatt	+
264	PBX1 intron 2	105296	105323	RSS12	cactttgacactcttgagcacaagaact	-
265	PBX1 intron 2	105394	105432	RSS23	cacattggcgcgaggagccggtcctggcagtgccaagct	-
266	PBX1 intron 2	105639	105666	RSS12	cacagtcttcctaccagactcccaagca	-
267	PBX1 intron 2	106326	106364	RSS23	cacaccctacttccctttcctgggttcatgcaacaaatc	+
268	PBX1 intron 2	107406	107444	RSS23	cagagagaggaaaatagcagagagaggaaaaagaaagtg	+
269	PBX1 intron 2	107515	107553	RSS23	cacaggcagaaagaaaacaccccttctgatcctcccccc	-
270	PBX1 intron 2	107531	107569	RSS23	cacacgggcgcagggacacaggcagaaagaaaacacccc	-
271	PBX1 intron 2	107816	107843	RSS12	caccgacagaaatcacaaaacaaatata	+
272	PBX1 intron 2	108247	108285	RSS23	catactgatggagggaaagggtaacgacaggggaaacta	-
273	PBX1 intron 2	108460	108487	RSS12	cactcacacaaaaccctcattccaaagg	-
274	PBX1 intron 2	109188	109215	RSS12	cacttaagctctcagctttgccattaaa	-
275	PBX1 intron 2	109374	109401	RSS12	cacgccgctacacatttatgtgatattc	-
276	PBX1 intron 2	110430	110468	RSS23	cacggcattcccataaaagtttcatctagttgagaagtt	-
277	PBX1 intron 2	110681	110719	RSS23	cacagagatgaaatgactatgagctgaaaacaagacgcc	+
278	PBX1 intron 2	110802	110840	RSS23	cacagacatggaggtttaccagggactgatggctaaaag	-
279	PBX1 intron 2	110960	110998	RSS23	cacatggacaaagaggaaggatgagttgactctaaacca	+
280	PBX1 intron 2	111236	111274	RSS23	cacagaattatccaggacattccatgcccatttatagtt	-
281	PBX1 intron 2	111551	111578	RSS12	cacccaccaccaagaggatctttaaact	+
282	PBX1 intron 2	111829	111856	RSS12	catcatctgtcaacccaggaccaataat	-
283	PBX1 intron 2	112952	112979	RSS12	cagatcctttcctgaacaagcagacacc	+
284	PBX1 intron 2	113346	113384	RSS23	cacttagctagcaagttagggggctgcaggctttgaacc	-
285	PBX1 intron 2	114040	114078	RSS23	caaagtgctgggattacaggcgtgagccaccgcgcccgg	+
286	PBX1 intron 2	114067	114105	RSS23	caccgcgcccggccttactgcttgacattttacaaaacg	+
287	PBX1 intron 2	114401	114428	RSS12	caccatgtgtccccaccctgcacacact	-
288	PBX1 intron 2	114755	114793	RSS23	cacaatgattccccactaccaatcacatcaagcctacca	-
289	PBX1 intron 2	114995	115022	RSS12	cagagagccttgtgtggatacaaacagg	-
290	PBX1 intron 2	114997	115024	RSS12	cacagagagccttgtgtggatacaaaca	-
291	PBX1 intron 2	115273	115300	RSS12	cacagcccatttaggggaaggctaaccc	+
292	PBX1 intron 2	116022	116060	RSS23	cactgtgatgtccacagcagccgatggtctcagtaactg	-
293	PBX1 intron 2	116054	116092	RSS23	cacagtgggagcagtctactgggtgggaggggtgctgtc	+
294	PBX1 intron 2	116415	116442	RSS12	cacaggtgcataaatttagaacataact	-
295	PBX1 intron 2	116603	116641	RSS23	caaactcctgctggtttttttttatttgtctgaaaactg	+
296	PBX1 intron 2	117130	117157	RSS12	caccaaacctcagcatcacacaatatac	-

297	PBX1 intron 2	117392	117430	RSS23	cagaatggggaaagttccactgaagcagattcagttcac	-
298	PBX1 intron 2	117673	117711	RSS23	caaagtgctgggattacaggcatgagccaccattcctgg	-
299	PBX1 intron 2	120244	120282	RSS23	cacattaacaaggtgatacagaagaaagacttagattcc	-
300	PBX1 intron 2	120487	120514	RSS12	caaaacaaaacaaaacaaacaaaatcca	-
301	PBX1 intron 2	120492	120519	RSS12	caaaacaaaacaaaacaaaacaaacaaa	-
302	PBX1 intron 2	120987	121014	RSS12	cacaaagtgtccagaaacagggaaaaca	-
303	PBX1 intron 2	121455	121482	RSS12	cacagtgccccagatctttttgccaata	-
304	PBX1 intron 2	122596	122634	RSS23	cacgggggaacaggcacctaatgttctccagcagaagct	-
305	PBX1 intron 2	123971	124009	RSS23	caaagtgctgggattagaggtgtgagccacgtgtctggc	-
306	PBX1 intron 2	124212	124250	RSS23	cactgcactccagctgggtgataaagtgagaccttgtct	+
307	PBX1 intron 2	125835	125862	RSS12	cattacggcaaacaccttcccaaagtga	+
308	PBX1 intron 2	126439	126466	RSS12	caaagagagccaagccagaacatcgatt	-
309	PBX1 intron 2	126481	126519	RSS23	cacggggagagagagatgaggacagggagaaagaaatga	-
310	PBX1 intron 2	126737	126764	RSS12	cagtaccatatgtcaccctgcaataaca	-
311	PBX1 intron 2	127962	128000	RSS23	caaagtgctggggttacaggcatgagccactgtgcccgg	+
312	PBX1 intron 2	128062	128100	RSS23	cacagtactctagtgtgatttgtattatgtcaggatgtg	-
313	PBX1 intron 2	128140	128167	RSS12	cactctaaaacacagttaaaattatcac	-
314	PBX1 intron 2	128524	128551	RSS12	caccaccatgatgccacaagtggaaaat	+
315	PBX1 intron 2	129531	129569	RSS23	cacagttttactaggttctaatttcatgaaagggaatcg	+
316	PBX1 intron 2	129722	129760	RSS23	cacagtgaccttggcgaacagtggtctcagggaaggtcc	-
317	PBX1 intron 2	129724	129762	RSS23	cacacagtgaccttggcgaacagtggtctcagggaaggt	-
318	PBX1 intron 2	129754	129792	RSS23	cactgtgtgtgccccacaggcctctgcaggaagaaagag	+
319	PBX1 intron 2	130447	130474	RSS12	caatgaagaacagatctgtccatagact	+
320	PBX1 intron 2	130501	130528	RSS12	caccatgacgtctgccttttcacaacgt	+
321	PBX1 intron 2	130501	130539	RSS23	caccatgacgtctgccttttcacaacgtgcagctaatca	+
322	PBX1 intron 2	130572	130599	RSS12	cagactgggagagcactttcacaactct	-
323	PBX1 intron 2	130715	130753	RSS23	cacagaactagagagactagtgattctaagatttctagc	+
324	PBX1 intron 2	132697	132735	RSS23	caaagtgaggcccatgtgcttgccattgatagtctgtca	+
325	PBX1 intron 2	133376	133414	RSS23	cacaacattcagtgaaactcaaaagatctaccacaatct	-
326	PBX1 intron 2	134454	134481	RSS12	cacaatcaggctaagtgatattatatcc	-
327	PBX1 intron 2	134487	134525	RSS23	cacatcacctcccccgaccaaagaaaagtcataaaagca	+
328	PBX1 intron 2	134993	135031	RSS23	cactctgtgcttattatttataccagtaagaccattatt	-
329	PBX1 intron 2	135023	135050	RSS12	cacagagtgtttcatacatatcaaagca	+
330	PBX1 intron 2	135023	135061	RSS23	cacagagtgtttcatacatatcaaagcaatttcgtacat	+
331	PBX1 intron 2	135395	135433	RSS23	cacaggctaggaagccagataaacatacaattagaatca	-
332	PBX1 intron 2	135653	135680	RSS12	cacagcccatgctatcagctatagcact	+
333	PBX1 intron 2	136765	136803	RSS23	cacaccctgtaataagcatctctgttccggcatgttgcg	-
334	PBX1 intron 2	137235	137273	RSS23	cactgtctgctcccgtgtcccccaacctagagcaaagcc	-
335	PBX1 intron 2	137955	137982	RSS12	cattgtgtttcaacacagaaaagaaaaa	-
336	PBX1 intron 2	138587	138614	RSS12	cacaaccttttggtctatattaaaaacc	+
337	PBX1 intron 2	138655	138693	RSS23	caaagtgcatgaaggggacatggagagaataaaaatctc	+
338	PBX1 intron 2	138945	138972	RSS12	catcataatgtgcacctgaacattcatg	+
339	PBX1 intron 2	139182	139220	RSS23	cacactcactcacactgaagcccaccttggtcaactcaa	-
340	PBX1 intron 2	139285	139323	RSS23	cactctacctgccactgtagaagcacacagtcaagcaca	-
341	PBX1 intron 2	139505	139532	RSS12	cagatacacacagacctacacaatgtca	-
342	PBX1 intron 2	139863	139890	RSS12	cactcagtttgatgtcaatatgatcacc	-
343	PBX1 intron 2	140057	140095	RSS23	cacaatgctacagcaccatcctgaagttgctcggctttc	-
344	PBX1 intron 2	140068	140095	RSS12	cacaatgctacagcaccatcctgaagtt	-
345	PBX1 intron 2	140196	140223	RSS12	cagagggacacaaacacatatgtacaaa	+
346	PBX1 intron 2	140566	140593	RSS12	cattcagagcaacttccctacaaatctc	+
347	PBX1 intron 2	140707	140745	RSS23	cacaaccagctttatttggtaaaatgctgcaatagatgc	+
348	PBX1 intron 2	140877	140904	RSS12	cacagaccaaggggtcttgtgggaaata	+
349	PBX1 intron 2	141616	141654	RSS23	caaaatactaccaaaaggagttcttgttttccccaactt	+
350	PBX1 intron 2	142598	142625	RSS12	cacagaattccagatcaaagtgccattc	-
351	PBX1 intron 2	142710	142737	RSS12	catagaattccaaaactcagcaatcaaa	-
352	PBX1 intron 2	143697	143735	RSS23	cacagtccctttcaactcaccactacagatggcaaactg	-
353	PBX1 intron 2	143809	143836	RSS12	cagaatatccaaggtctcataaaagatt	-
354	PBX1 intron 2	144050	144077	RSS12	cagtattgtggctaatgagactaacacc	+
355	PBX1 intron 2	144052	144090	RSS23	cacacacctcagtggtgttagtctcattagccacaatac	-
356	PBX1 intron 2	144068	144106	RSS23	cacacacacacacacacacacctcagtggtgttagtc	-

357	PBX1 intron 2	144832	144870	RSS23	cagagcgaggcagagtgactaagtgactgctggaatcct	+
358	PBX1 intron 2	145189	145216	RSS12	cactctcttgagtgctgtgttcattatt	-
359	PBX1 intron 2	145673	145700	RSS12	cagtgtcctagtccttgaggtgtacaca	+
360	PBX1 intron 2	146054	146081	RSS12	caaagctgggtccattgaaacagaaagc	+
361	PBX1 intron 2	146260	146298	RSS23	caccaagacatttcacgcagaccacccactgcaatttct	+
362	PBX1 intron 2	148211	148249	RSS23	caaagtgctgggattacaggcgtgagccactgcgcccgg	+
363	PBX1 intron 2	148385	148423	RSS23	cacaaaatcccacatgcgtgggcttgttccctgcaagct	+
364	PBX1 intron 2	149855	149882	RSS12	cagagaagtttctgcctcttggtaaaac	+
365	PBX1 intron 2	150384	150422	RSS23	cacacagattgaagtaggcacagaaggcagcagaaagca	+
366	PBX1 intron 2	150720	150747	RSS12	cacaacatttggaaccctccccagagct	-
367	PBX1 intron 2	151105	151132	RSS12	cacagggtaataagtgtttgcaagaact	-
368	PBX1 intron 2	152814	152841	RSS12	caagatgttttcagatcatacaaaaaca	+
369	PBX1 intron 2	152819	152846	RSS12	cacattgtttttgtatgatctgaaaaca	-
370	PBX1 intron 2	152874	152901	RSS12	cagtgggtggtgtgtacagacatacaca	-
371	PBX1 intron 2	152880	152918	RSS23	cactgccttgcatagatcagtgggtggtgtgtacagaca	-
372	PBX1 intron 2	153376	153403	RSS12	cacacacacacacacacacacacacaca	+
373	PBX1 intron 2	153378	153405	RSS12	cacacacacacacacacacacacacaca	+
374	PBX1 intron 2	153380	153407	RSS12	cacacacacacacacacacacacacaca	+
375	PBX1 intron 2	153382	153409	RSS12	cacacacacacacacacacacacacaca	+
376	PBX1 intron 2	153384	153411	RSS12	cacacacacacacacacacacacacaca	+
377	PBX1 intron 2	153386	153413	RSS12	cacacacacacacacacacacacacaca	+
378	PBX1 intron 2	153386	153424	RSS23	cacacacacacacacacacacacacacacacacagaaat	+
379	PBX1 intron 2	153388	153415	RSS12	cacacacacacacacacacacacacaca	+
380	PBX1 intron 2	153388	153426	RSS23	cacacacacacacacacacacacacacacacagaaatac	+
381	PBX1 intron 2	153390	153417	RSS12	cacacacacacacacacacacacacaca	+
382	PBX1 intron 2	153390	153428	RSS23	cacacacacacacacacacacacacacacagaaatacac	+
383	PBX1 intron 2	153392	153419	RSS12	cacacacacacacacacacacacacaca	+
384	PBX1 intron 2	153396	153434	RSS23	cacacacacacacacacacacacagaaatacacgaagag	+
385	PBX1 intron 2	153398	153425	RSS12	cacacacacacacacacacacagaaata	+
386	PBX1 intron 2	153400	153427	RSS12	cacacacacacacacacacagaaataca	+
387	PBX1 intron 2	153400	153438	RSS23	cacacacacacacacacacagaaatacacgaagagaaga	+
388	PBX1 intron 2	153404	153442	RSS23	cacacacacacacacagaaatacacgaagagaagaaaaa	+
389	PBX1 intron 2	153408	153446	RSS23	cacacacacacagaaatacacgaagagaagaaaaaaaaa	+
390	PBX1 intron 2	153410	153448	RSS23	cacacacacagaaatacacgaagagaagaaaaaaaaaga	+
391	PBX1 intron 2	153414	153452	RSS23	cacacagaaatacacgaagagaagaaaaaaaaagaatct	+
392	PBX1 intron 2	153954	153981	RSS12	catattcttaaacaggacctcataaaac	-
393	PBX1 intron 2	153993	154031	RSS23	cacaacacaccacaggcaaataaaagtctccaaagaaat	-
394	PBX1 intron 2	154042	154080	RSS23	caccctgtggccaagaagagcctctctcttcccaaagag	-
395	PBX1 intron 2	154778	154805	RSS12	cacagagttggaagtcagatccaagtct	+
396	PBX1 intron 2	154778	154816	RSS23	cacagagttggaagtcagatccaagtctgattcctaaac	+
397	PBX1 intron 2	154782	154820	RSS23	cacagtttaggaatcagacttggatctgacttccaactc	-
398	PBX1 intron 2	155200	155238	RSS23	cacagaaccattttgaacactccatttgtcacccaaatc	-
399	PBX1 intron 2	155403	155441	RSS23	cacaaagtcaaagagtcaaaagcattctgagtctccggc	-
400	PBX1 intron 2	155414	155441	RSS12	cacaaagtcaaagagtcaaaagcattct	-
401	PBX1 intron 2	155442	155480	RSS23	cacaatgataagagtctttcaatatctccaccatattca	-
402	PBX1 intron 2	155453	155480	RSS12	cacaatgataagagtctttcaatatctc	-
403	PBX1 intron 2	155825	155852	RSS12	cacagaaatgccagtctgtgagagagaa	+
404	PBX1 intron 2	157123	157161	RSS23	cacagtagaaaatgtcagtcatatttccaagaatctctc	-
405	PBX1 intron 2	157579	157617	RSS23	cactgtaaatatacctcaggaaggaaaccgcagaaaccc	-
406	PBX1 intron 2	158058	158085	RSS12	caccgagatttggagcagtgccctaaac	-
407	PBX1 intron 2	158098	158136	RSS23	cacacacacacacacactacactgccacccaatttta	-
408	PBX1 intron 2	159508	159535	RSS12	caccctgggcagttgctcttcaaaaagg	-
409	PBX1 intron 2	162012	162050	RSS23	cacacaggcagacagatcaagccgctatgtgagggacaa	-
410	PBX1 intron 2	163405	163443	RSS23	cacagtgtgaggtacagagctgcttttactagtaaaaca	-
411	PBX1 intron 2	163578	163605	RSS12	cactaagacaaaagctgagccccaattc	-
412	PBX1 intron 2	163639	163677	RSS23	cacagaaaaccaaagcgaaggtttcttcctgcattatgg	-
413	PBX1 intron 2	164034	164061	RSS12	cactgtgtcacattaataaaactaaatc	+
414	PBX1 intron 2	164034	164072	RSS23	cactgtgtcacattaataaaactaaatcaggttataatt	+
415	PBX1 intron 2	164734	164772	RSS23	caaagtgataggattacaggcatgagccaccacgcccag	-
416	PBX1 intron 2	165098	165125	RSS12	cacatttgtgcagaaccttttcaacact	-

417	PBX1 intron 2	165226	165264	RSS23	cacaggcagcagatagcccattattcagaattataatgt	+
418	PBX1 intron 2	165523	165561	RSS23	cacagctctatgaagaaaaataacaggccatttaaactg	-
419	PBX1 intron 2	166018	166045	RSS12	cacaaggataaatagcagaaggtaattc	-
420	PBX1 intron 2	166664	166702	RSS23	cactggggcaggtttagaaactaagtctgtttctaaata	-
421	PBX1 intron 2	167675	167713	RSS23	catagaggggtagcattcatcagaataaggtcagaaaaa	+
422	PBX1 intron 2	167728	167766	RSS23	cacacacacacataaacacggaaagcaatcgcaaaaaac	-
423	PBX1 intron 2	168143	168170	RSS12	cacaatatgagggagtcccttcatatcc	-
424	PBX1 intron 2	168218	168245	RSS12	cactgtgaatgccgttgaaagggttact	+
425	PBX1 intron 2	168819	168857	RSS23	caccaacacagaatgttggcttcactagctcacaaagcc	-
426	PBX1 intron 2	169839	169877	RSS23	catgcagagccagtctgaagtcagaactcaacagaaaca	-
427	PBX1 intron 2	169861	169888	RSS12	cagactggctctgcatggatctcattct	+
428	PBX1 intron 2	169890	169928	RSS23	caaacaccctgagagtgatgggatccctcaacaagaatg	-
429	PBX1 intron 2	169994	170032	RSS23	cacaaggacagaatgccattatcaacacaatgggtccct	+
430	PBX1 intron 2	170715	170742	RSS12	caaaatgaatccacacttacacaacata	-
431	PBX1 intron 2	170757	170784	RSS12	cacagtcagcaaacatgtagtgaaaaat	-
432	PBX1 intron 2	172051	172078	RSS12	cacatacatgtaggcacagaccaatacc	-
433	PBX1 intron 2	172644	172671	RSS12	cacaatgactcctgagcaaacaaactgc	-
434	PBX1 intron 2	172650	172688	RSS23	cacatccatagacaagccacaatgactcctgagcaaaca	-
435	PBX1 intron 2	173473	173511	RSS23	cacagtactctcactagctcttctctacttgattctccc	-
436	PBX1 intron 2	173679	173706	RSS12	cactgaatcctatctcatctcataatcc	-
437	PBX1 intron 2	174427	174465	RSS23	caaagtgctgggattacaggcgtgagccactgtacctgg	+
438	PBX1 intron 2	174780	174807	RSS12	cacaataaaatactactcagaaacaaca	-
439	PBX1 intron 2	174798	174825	RSS12	cacattgtggtctattcccacaataaaa	-
440	PBX1 intron 2	175265	175292	RSS12	caatatatttaatttcataagaaacaac	+
441	PBX1 intron 2	175305	175332	RSS12	cacagtggttatgcctctaaccccttct	+
442	PBX1 intron 2	176072	176110	RSS23	cacaagctgtgagagagaaccaagccctgtgtagaacac	-
443	PBX1 intron 2	176416	176454	RSS23	cacacacacacatgcacaaccacttcatgaaccctgcag	-
444	PBX1 intron 2	176422	176460	RSS23	cacacacacacacacacatgcacaaccacttcatgaacc	-
445	PBX1 intron 2	176428	176466	RSS23	cacacacacacacacacacacacatgcacaaccacttca	-
446	PBX1 intron 2	176433	176460	RSS12	cacacacacacacacacatgcacaacca	-
447	PBX1 intron 2	176434	176472	RSS23	cacacacacacacacacacacacacacatgcacaacc	-
448	PBX1 intron 2	176437	176464	RSS12	cacacacacacacacacacacatgcaca	-
449	PBX1 intron 2	176443	176470	RSS12	cacacacacacacacacacacacacaca	-
450	PBX1 intron 2	176445	176472	RSS12	cacacacacacacacacacacacacaca	-
451	PBX1 intron 2	176447	176474	RSS12	cacacacacacacacacacacacacaca	-
452	PBX1 intron 2	176449	176476	RSS12	cacacacacacacacacacacacacaca	-
453	PBX1 intron 2	176451	176478	RSS12	cacacacacacacacacacacacacaca	-
454	PBX1 intron 2	176455	176482	RSS12	catacacacacacacacacacacacaca	-
455	PBX1 intron 2	176498	176525	RSS12	cacattagtgttcatttgtctgagaact	+
456	PBX1 intron 2	176744	176782	RSS23	catttagatcaaattctgtttcttcttcagacataaaat	-
457	PBX1 intron 2	177567	177594	RSS12	cacatctagtaacagttgatcaaatatc	-
458	PBX1 intron 2	177788	177815	RSS12	cacaatctataccatatcgaggataggc	-
459	PBX1 intron 2	178346	178384	RSS23	cacagtggtcttcatctagagatcataagtaagatgatg	-
460	PBX1 intron 2	178403	178441	RSS23	cacacacacacactcactcatacatgctcactccaacct	-
461	PBX1 intron 2	178413	178451	RSS23	cacacacacacacacacacactcactcatacatgctc	-
462	PBX1 intron 2	178416	178443	RSS12	cacacacacacacactcactcatacatg	-
463	PBX1 intron 2	178430	178457	RSS12	cacacacacacacacacacacacacaca	-
464	PBX1 intron 2	178432	178459	RSS12	cacacacacacacacacacacacacaca	-
465	PBX1 intron 2	178434	178461	RSS12	cacacacacacacacacacacacacaca	-
466	PBX1 intron 2	178436	178463	RSS12	cacacacacacacacacacacacacaca	-
467	PBX1 intron 2	178951	178989	RSS23	caaagtgctgggattacaggcgtgagccaccactcctgg	-
468	PBX1 intron 2	179748	179786	RSS23	cagggccagaaacagtgatggggctaagccagagaaacc	+
469	PBX1 intron 2	181378	181405	RSS12	cagggacataagggtcaattcaaaaaag	+
470	PBX1 intron 2	183200	183238	RSS23	caaaacttagccgggcgtggtggcacatgcctgtaatcc	-
471	PBX1 intron 2	183745	183772	RSS12	caatctgtttaagcctcaattcctcacc	-
472	PBX1 intron 2	184189	184227	RSS23	cacacaaagtcaatggctgttctaacctaaagtctatct	-
473	PBX1 intron 2	184217	184255	RSS23	cagagagacacagttacttccttaaggacacacaaagtc	-
474	PBX1 intron 2	184254	184281	RSS12	cagtgtgcccgtggtcattcaagattca	-
475	PBX1 intron 2	184696	184734	RSS23	cacaatggctaaacttgatcattttggagtaagaatagt	-
476	PBX1 intron 2	184901	184928	RSS12	caccataaatagtaattgtacaatacaa	+

477	PBX1 intron 2	185466	185493	RSS12	cacattgcatcagacctcaaatgaagac	+
478	PBX1 intron 2	185466	185504	RSS23	cacattgcatcagacctcaaatgaagactggaacatatc	+
479	PBX1 intron 2	186279	186317	RSS23	caaagggtccaagccttgctaataacagtgggttaatca	-
480	PBX1 intron 2	186485	186523	RSS23	caccctgtctgatggtgattaccagcatctgacagatca	+
481	PBX1 intron 2	186811	186849	RSS23	cacacacacacaccaactagcccccagtttgaaagct	-
482	PBX1 intron 2	186817	186855	RSS23	cacacacacacacacacaccaactagcccccagtttg	-
483	PBX1 intron 2	186823	186861	RSS23	cacacacacacacacacacacacaccaactagccccc	-
484	PBX1 intron 2	186829	186867	RSS23	cacacacacacacacacacacacacacaccaacta	-
485	PBX1 intron 2	186830	186857	RSS12	cacacacacacacacacacaccaact	-
486	PBX1 intron 2	186834	186861	RSS12	cacacacacacacacacacacacacc	-
487	PBX1 intron 2	186836	186863	RSS12	cacacacacacacacacacacacaca	-
488	PBX1 intron 2	186838	186865	RSS12	cacacacacacacacacacacacaca	-
489	PBX1 intron 2	186840	186867	RSS12	cacacacacacacacacacacacaca	-
490	PBX1 intron 2	186842	186869	RSS12	cacacacacacacacacacacacaca	-
491	PBX1 intron 2	186844	186871	RSS12	cacacacacacacacacacacacaca	-
492	PBX1 intron 2	187725	187763	RSS23	caaagtgctgggattacaggcatgagtcaccgcacccag	-
493	PBX1 intron 2	188036	188063	RSS12	cacattcataaaagactgagagtaaata	-
494	PBX1 intron 2	188071	188109	RSS23	cagtctgtgacaatattacatgaagcctgtggaaaactc	-
495	PBX1 intron 2	188722	188760	RSS23	cacacaggtacagacagccagtttttagaagcagtgttt	-
496	PBX1 intron 2	188883	188921	RSS23	cacacacacacacacacacacacacaaaaccttaaaggg	-
497	PBX1 intron 2	188890	188917	RSS12	cacacacacacacacacacacaaaacct	-
498	PBX1 intron 2	188891	188929	RSS23	cacacacacacacacacacacacacacacacacaaaacc	-
499	PBX1 intron 2	188892	188919	RSS12	cacacacacacacacacacacacaaaac	-
500	PBX1 intron 2	188896	188923	RSS12	cacacacacacacacacacacacaca	-
501	PBX1 intron 2	188898	188925	RSS12	cacacacacacacacacacacacaca	-
502	PBX1 intron 2	188900	188927	RSS12	cacacacacacacacacacacacaca	-
503	PBX1 intron 2	188902	188929	RSS12	cacacacacacacacacacacacaca	-
504	PBX1 intron 2	188904	188931	RSS12	cacacacacacacacacacacacaca	-
505	PBX1 intron 2	188906	188933	RSS12	cacacacacacacacacacacacaca	-
506	PBX1 intron 2	188908	188935	RSS12	cacacacacacacacacacacacaca	-
507	PBX1 intron 2	189240	189278	RSS23	cactgtggggcaacaggacctaccacttaattagattat	-
508	PBX1 intron 2	189272	189310	RSS23	cacagtgtcttttggtgtattaggggaataaagtgatct	+
509	PBX1 intron 2	189630	189657	RSS12	cattataaaacacacacatgcacagact	-
510	PBX1 intron 2	189654	189692	RSS23	cacaacccttccaggttttattcttttgaaactttcatt	-
511	PBX1 intron 2	190354	190392	RSS23	cacagtggccctggggaaggagaagccactctcatttcc	-
512	PBX1 intron 2	190666	190704	RSS23	cacgctgctcctcgatacatactaaatgacaacaaagag	-
513	PBX1 intron 2	190776	190814	RSS23	cactatcacccagagattaggacaggctctaagtccccc	-
514	PBX1 intron 2	190803	190841	RSS23	cacataggcagcagggcagtgggcacacactatcaccca	-
515	PBX1 intron 2	191324	191362	RSS23	cacgtacacacacaccccctctgttttagaacaataaact	-
516	PBX1 intron 2	191354	191392	RSS23	cacacacacacacacgcacgcacgtgcacgcacgtacac	-
517	PBX1 intron 2	191517	191555	RSS23	cactgtgttgtgttcttttaatcaaaaagcttaaaataa	+
518	PBX1 intron 2	191870	191908	RSS23	cactgcactccagcctgggcaagaagggtgaaaataccc	-
519	PBX1 intron 2	192740	192767	RSS12	cacagagctagctggcatacaattaata	+
520	PBX1 intron 2	192939	192977	RSS23	cacactgccctatgtaacccaagtccttgtcacaaaagg	+
521	PBX1 intron 2	192969	193007	RSS23	cacaaaagggctgtctgattttttttcccctttaaaatc	+
522	PBX1 intron 2	193252	193279	RSS12	catagaagaaaaaggcatcacataaatt	+
523	PBX1 intron 2	194467	194494	RSS12	cacattaggttgggttgacccagtaaaa	+
524	PBX1 intron 2	194813	194851	RSS23	cacagtaattttccctccttgattctacaacttctaccc	+
525	PBX1 intron 2	196589	196627	RSS23	cacacagtaagatatgactgcttgacctccacatggagc	+
526	PBX1 intron 2	198017	198044	RSS12	caccctcctccacacaaacacatttaaa	-
527	PBX1 intron 2	198391	198429	RSS23	caaagtgctgggattacaggcgtgagccaccgcgcccgg	+
528	PBX1 intron 2	198479	198517	RSS23	cacactgatccaaataaaggtatagcctgaactaaatag	-
529	PBX1 intron 2	199898	199925	RSS12	cagagtggctccatagaccacaaaaaac	+
530	PBX1 intron 2	200635	200662	RSS12	cacaatcctataaaccattctattaaag	-
531	PBX1 intron 2	200789	200816	RSS12	caaattcccaagtacctgggcatatact	+
532	PBX1 intron 2	201285	201312	RSS12	caaaataagacaactaaattatgagact	-
533	PBX1 intron 2	202476	202503	RSS12	cacagtgacacaagtttccatggtaccc	-
534	PBX1 intron 2	202525	202552	RSS12	cagggtgtgaaatgccccttaaaatact	+
535	PBX1 intron 2	203248	203286	RSS23	cacagctagtgagaagcagagacttggccttatcccacg	+
536	PBX1 intron 2	204483	204521	RSS23	cacagaaaaacagatgggatagaaatttacacaaaaatc	+

537	PBX1 intron 2	204573	204600	RSS12	cacagggtatttaaatagaacaagattt	+
538	PBX1 intron 2	205017	205055	RSS23	cagactacccatcctgttctgaattccaacacaagaagc	+
539	PBX1 intron 2	205062	205100	RSS23	cacagaggggcacagggcctgcaaagcaatccagcccct	+
540	PBX1 intron 2	205224	205262	RSS23	cacacaatggaaagccgttgcaatctataaatccaagtc	-
541	PBX1 intron 2	206147	206185	RSS23	caaagtgagacttcatcatgtgaagggcagaaaagagtg	-
542	PBX1 intron 2	206339	206366	RSS12	cacagtaataaaagttggctcaaggtcc	-
543	PBX1 intron 2	208347	208385	RSS23	cacactgggacaaactattttttattttgggtaatattt	+
544	PBX1 intron 2	208410	208437	RSS12	cactgtgtcacatgagctgtgtttaaca	-
545	PBX1 intron 2	208862	208889	RSS12	cactgtgatggaaagtatttcataatta	-
546	PBX1 intron 2	209713	209751	RSS23	cacaaaatgcaagaaggaaccaaaatctaccaagaatta	-
547	PBX1 intron 2	209724	209751	RSS12	cacaaaatgcaagaaggaaccaaaatct	-
548	PBX1 intron 2	209751	209789	RSS23	cagagggctgagaacaactgctcttagtgggacaaagcc	-
549	PBX1 intron 2	209933	209960	RSS12	cacacacacacacacacacacacacaca	+
550	PBX1 intron 2	209935	209962	RSS12	cacacacacacacacacacacacacaca	+
551	PBX1 intron 2	209941	209979	RSS23	cacacacacacacacacacacagagtttttcttgatatg	+
552	PBX1 intron 2	209943	209981	RSS23	cacacacacacacacacacagagtttttcttgatatgct	+
553	PBX1 intron 2	209947	209985	RSS23	cacacacacacacacagagtttttcttgatatgctgtct	+
554	PBX1 intron 2	210614	210641	RSS12	cactaacatggggagtcgtagaaaaata	-
555	PBX1 intron 2	210901	210939	RSS23	cacagggttctttgggaagcttcaaaaaattcaaaagtc	+
556	PBX1 intron 2	211784	211822	RSS23	cactccacttctcacccctcccagatcagaggtgaagcc	-
557	PBX1 intron 2	211795	211822	RSS12	cactccacttctcacccctcccagatca	-
558	PBX1 intron 2	212157	212184	RSS12	cacactcaaagatgttttcttcaaagcc	+
559	PBX1 intron 2	212299	212326	RSS12	cactgggcagacggttataacaaaatgc	-
560	PBX1 intron 2	213556	213594	RSS23	cacacaaatgtaaaaccacaagatttactgcagagattc	-
561	PBX1 intron 2	213577	213604	RSS12	cacacacacacacacaaatgtaaaacca	-
562	PBX1 intron 2	213578	213616	RSS23	cacacacacacacacacacacacacacaaatgtaaaacc	-
563	PBX1 intron 2	213579	213606	RSS12	cacacacacacacacacaaatgtaaaac	-
564	PBX1 intron 2	213583	213610	RSS12	cacacacacacacacacacacaaatgta	-
565	PBX1 intron 2	213584	213622	RSS23	cacacacacacacacacacacacacacacacacaaatgt	-
566	PBX1 intron 2	213585	213612	RSS12	cacacacacacacacacacacacaaatg	-
567	PBX1 intron 2	213589	213616	RSS12	cacacacacacacacacacacacacaca	-
568	PBX1 intron 2	213591	213618	RSS12	cacacacacacacacacacacacacaca	-
569	PBX1 intron 2	213593	213620	RSS12	cacacacacacacacacacacacacaca	-
570	PBX1 intron 2	213595	213622	RSS12	cacacacacacacacacacacacacaca	-
571	PBX1 intron 2	213597	213624	RSS12	cacacacacacacacacacacacacaca	-
572	PBX1 intron 2	213845	213883	RSS23	caaagtgctgggattacaggcatgagccaccatgcccag	-
573	PBX1 intron 2	214061	214099	RSS23	cactctgtagcccagggtggagtgcagtggccagatctt	-
574	PBX1 intron 2	214093	214131	RSS23	cagagtgagaccctgcctcaaacaacaacaacaaaatct	+
575	PBX1 intron 2	214265	214303	RSS23	cactgtcacaggactacagtagtagcaaacaaaggaggg	+
576	PBX1 intron 2	215053	215091	RSS23	caaagtgttgggattacaggtgtgagccactgcacctgg	-
577	PBX1 intron 2	215460	215498	RSS23	cacattggccttcatcccgtgaaactctgccagctgcta	+
578	PBX1 intron 2	215539	215577	RSS23	cacagctgcctccaattgtagcaaggttgatagcaagca	-
579	PBX1 intron 2	215590	215617	RSS12	caccctgagtgaagtcaactgcagaact	+
580	PBX1 intron 2	215636	215663	RSS12	cactttgactcccagtacctcagtttcc	+
581	PBX1 intron 2	215806	215844	RSS23	cactgcaagtcaaaggaggtaatttttgacaatggattt	+
582	PBX1 intron 2	216091	216129	RSS23	cacaaagtgtgactgggctgcacaattttgccaacattg	-
583	PBX1 intron 2	216567	216605	RSS23	cacagtgagcaggaaagccagacacctcttcagagaaat	+
584	PBX1 intron 2	216834	216861	RSS12	cacagaaagaaggaatggtccagagacc	+
585	PBX1 intron 2	216854	216881	RSS12	cagagacccccctgctctccccaaattcc	+
586	PBX1 intron 2	218464	218491	RSS12	caaggtctctttgatctgttctaaaacc	+
587	PBX1 intron 2	218604	218631	RSS12	cactgtgcaatactctacatgaaaacac	+
588	PBX1 intron 2	220063	220101	RSS23	cacgcacacaaatgcacacagacacccccacccccacc	-
589	PBX1 intron 2	221174	221212	RSS23	caaaacctaacccagggaaaaagatgacaagaaaaactt	+
590	PBX1 intron 2	222212	222250	RSS23	cacacaaatcttcagattttcagttgttatccacccacc	+
591	PBX1 intron 2	222729	222767	RSS23	caagttgtaccagagccagcatgctgttgggtagaaggc	-
592	PBX1 intron 2	223064	223091	RSS12	cagaggcttagagatcattttcggctct	+
593	PBX1 intron 2	223321	223348	RSS12	caccgatactctggccaagaagaaaaca	-
594	PBX1 intron 2	223350	223388	RSS23	cacacacacacacacacagtctcttctgatgcccaaggc	-
595	PBX1 intron 2	223356	223394	RSS23	cacacacacacacacacacacacagtctcttctgatgcc	-
596	PBX1 intron 2	223357	223384	RSS12	cacacacacacacagtctcttctgatgc	-

597	PBX1 intron 2	223371	223398	RSS12	cacacacacacacacacacacacacaca	-
598	PBX1 intron 2	223373	223400	RSS12	cacacacacacacacacacacacacaca	-
599	PBX1 intron 2	223375	223402	RSS12	cacacacacacacacacacacacacaca	-
600	PBX1 intron 2	223377	223404	RSS12	cacacacacacacacacacacacacaca	-
601	PBX1 intron 2	223379	223406	RSS12	cacacacacacacacacacacacacaca	-
602	PBX1 intron 2	223381	223408	RSS12	cacacacacacacacacacacacacaca	-
603	PBX1 intron 2	223383	223410	RSS12	cacacacacacacacacacacacacaca	-
604	PBX1 intron 2	223631	223658	RSS12	cactgaattacatcaccttacaaaacat	-
605	PBX1 intron 2	223654	223681	RSS12	cagtgcgtgcaagtactttatgaacaaa	+
606	PBX1 intron 2	223818	223856	RSS23	cacaaacctccatgcagagtgttctattgcccaggagct	-
607	PBX1 intron 2	223906	223944	RSS23	cacaccgttgggaggctaccttgtgcctttccccagacc	+
608	PBX1 intron 2	224291	224318	RSS12	cacacaaggagaaaaacaaaccaaaccc	-
609	PBX1 intron 2	224933	224971	RSS23	catggtgctgcagagataaaaaggagcttttaaataatc	+
610	PBX1 intron 2	225472	225510	RSS23	cacataaataggtccccaatcagagttctgaagaaaaca	-
611	PBX1 intron 2	227125	227163	RSS23	cacaacactaccacgtcatgatcttctctcttcaaacct	-
612	PBX1 intron 2	227612	227639	RSS12	cactgtccccctggggctcaccagatgc	+
613	PBX1 intron 2	227986	228013	RSS12	caccaagggtcctggtgttttcataaag	+
614	PBX1 intron 2	228099	228126	RSS12	cacccttgtttccaacaggacagagtcc	+

Table S7: Potential cryptic recombination signal sites in *TCF3* intron 16

#	query	begin	end	type	sequence	orientation
1	TCF3 intron 16	68	106	RSS12	cacggtgacacctgccctgggctcccaccctgaccccca	-
2	TCF3 intron 16	391	429	RSS12	caccaccttgtggcctcatgggccaccatcttcaaagcc	-
3	TCF3 intron 16	538	576	RSS12	cactctgctccaaccccacgggtctcctggctgttcctc	-
4	TCF3 intron 16	865	892	RSS12	caccctcaagtcctgtccatcacaaatc	-
5	TCF3 intron 16	1051	1089	RSS12	cacagggccaggacagcgcccacaatgaccccaccttct	-
6	TCF3 intron 16	1621	1659	RSS12	cacagtgccccaaatgaatgcggccactctgcattcagc	-
7	TCF3 intron 16	1689	1727	RSS12	cacgctagtgggacgccgtgttcatgaagctcaagaaca	-
8	TCF3 intron 16	1766	1804	RSS12	cacaatgggaccccagccgctcacaccacacatgtaccc	-
9	TCF3 intron 16	2446	2484	RSS12	caaagtgctgggatcacaggcgtgagtcagccactgtgc	+
10	TCF3 intron 16	2640	2678	RSS12	cactgtactccagcctgggtgacaaagcaagactccgtc	-
11	TCF3 intron 16	2651	2678	RSS12	cactgtactccagcctgggtgacaaagc	-
12	TCF3 intron 16	2883	2921	RSS12	cacagtggctcacaccagctcacacctgtaatgccagca	-

Figure S8: Two-dimensional DNAfold model of the *TCF3* hotspot region

```
1       10        20        30        40        50        60        70        80
•        •         •         •         •         •         •         •         •
GCCTTCCACCAGCCCAGGAATCCTGCCTGCTTTCCAGGCAGACTTTCCAAGTACCTTGATTCTATCACTCCTAGGCCAGG
81      90        100       110       120       130       140       150       160
•        •         •         •         •         •         •         •         •
GCATCTCACCGCAGCGGCTCTGCCCCCAGGGGACACTGGGTGATGTCTGGGGACATCTACAGTTGTCAGGGCTGAGGGGA
161     170       180       190       200
•        •         •         •         •
GCTCCTGGCATGGAGTGGGTGGGGGCCAGGGATGCTGCTC
```

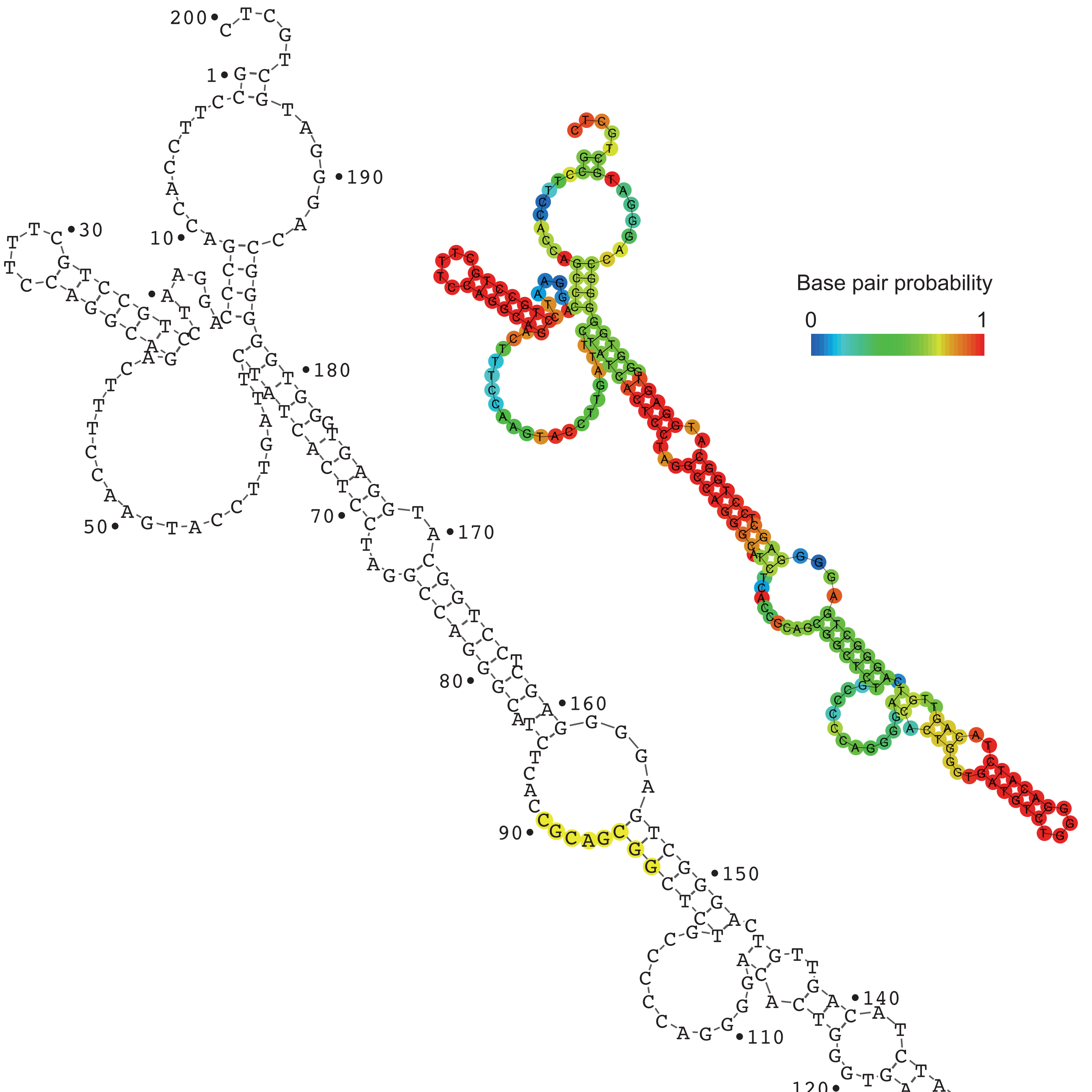

Ref.:
Gruber AR, Lorenz R, Bernhart SH, Neuböck R, Hofacker IL.
The Vienna RNA Websuite. Nucleic Acids Research 36, suppl 2, 12008, W70-W74,
DOI: 10.1093/nar/gkn188

Figure S9: Original gel images from Figure 1

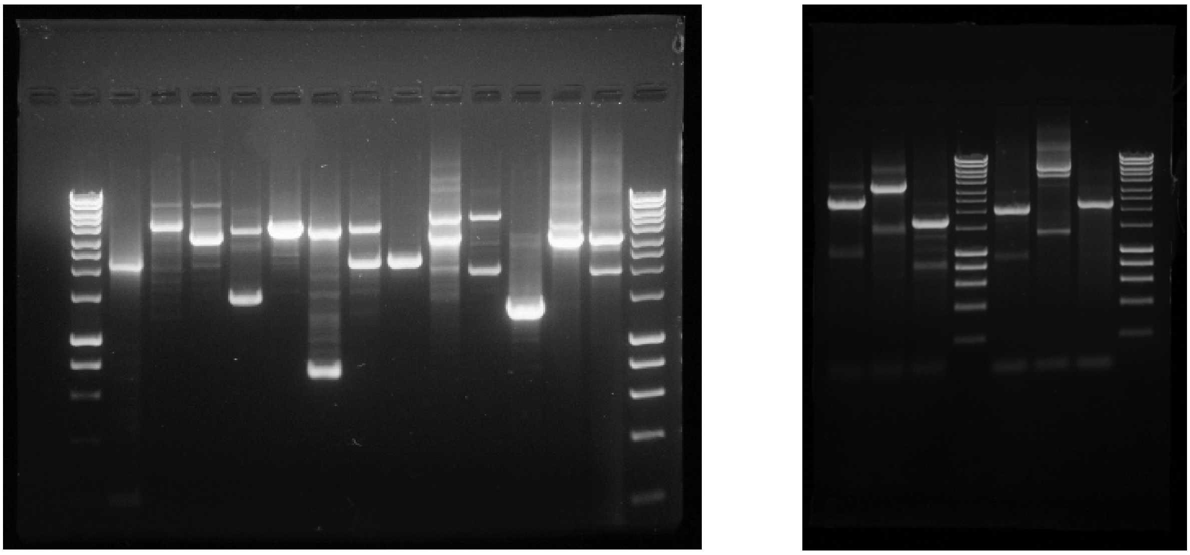

Supplement: Supplementary file 1 — Supplementary Information. [file 41598_2023_42294_MOESM1_ESM.pdf]
